# Artificial genetic polymers against human pathologies

**DOI:** 10.1186/s13062-022-00353-7

**Published:** 2022-12-06

**Authors:** Gleb S. Ivanov, Vyacheslav G. Tribulovich, Nikolay B. Pestov, Temitope I. David, Abdul-Saleem Amoah, Tatyana V. Korneenko, Nikolai A. Barlev

**Affiliations:** 1 Institute of Cytology, Tikhoretsky Ave 4, Saint Petersburg, Russia 194064; 2 St. Petersburg State Technological Institute (Technical University), Saint Petersburg, Russia 190013; 3 Chumakov Federal Scientific Center for Research and Development of Immune-and-Biological Products, Moscow, Russia 108819; 4 Phystech School of Biological and Medical Physics, Moscow Institute of Physics and Technology, Dolgoprudny, Moscow Region Russia 141701; 5 Shemyakin-Ovchinnikov Institute of Bioorganic Chemistry, Moscow, Russia 117997; 6 Institute of Biomedical Chemistry, Moscow, Russia 119121б; 7 School of Medicine, Nazarbayev University, 010000 Astana, Kazakhstan

**Keywords:** Locked nucleic acids, Peptide nucleic acids, Xenonucleic acids

## Abstract

Originally discovered by Nielsen in 1991, peptide nucleic acids and other artificial genetic polymers have gained a lot of interest from the scientific community. Due to their unique biophysical features these artificial hybrid polymers are now being employed in various areas of theranostics (therapy and diagnostics). The current review provides an overview of their structure, principles of rational design, and biophysical features as well as highlights the areas of their successful implementation in biology and biomedicine. Finally, the review discusses the areas of improvement that would allow their use as a new class of therapeutics in the future.

**Supplementary Information:**

The online version contains supplementary material available at 10.1186/s13062-022-00353-7.

## Background

To date, a huge number of modifications to nucleic acids have been investigated, and from this a very broad class of synthetic polymers—Artificial Genetic Polymers—has emerged. The meaning of the term AGP overlaps with that of xenonucleic acids (XNA), which are usually defined as nucleic acids with backbones significantly different from conventional (deoxy)ribose phosphodiesters. However, the definition of AGP is more convenient as AGPs include both small modifications such as 2’-methoxy-RNA, and radically different backbones such as PNA, which even is devoid of a notable feature of natural DNA/RNA—its acidity. The term “nucleic acid mimics” is somewhat misleading and should be reserved for entities of a completely different chemical nature.

A deep and comprehensive analysis of new classes of nucleic acids was undertaken by Anosova et al.[1], who suggested the possibility of storing some of the cell’s genetic information on more inert carriers than natural nucleic acids. Since then, a number of excellent reviews have appeared [2–9], alongside some especially exciting ones devoted to the prospects of creating orthogonal replication competent systems or xeno-lives [10–13] which include completely different two-base strands with dynamic covalent interactions between boronic acids (BAs) and catechols (CAs) as synthetic nucleobase analogs [14].

In general, AGPs have several properties making them a powerful tool for probing intricate biological processes and medical interventions therein. The most advantageous AGPs not only bind specifically to classical nucleic acids in a complementarity dependent manner, but allow the formation of more stable complexes, for example, lacking high sensitivity to ionic strength or pH, and, more importantly, they provide remarkable protection against biodegradation. Here we review some of these critical features of AGPs and outline their certain attractive perspectives with the special focus on their applications in oncology.

## Major structural types of AGPs

**Fig. 1:**
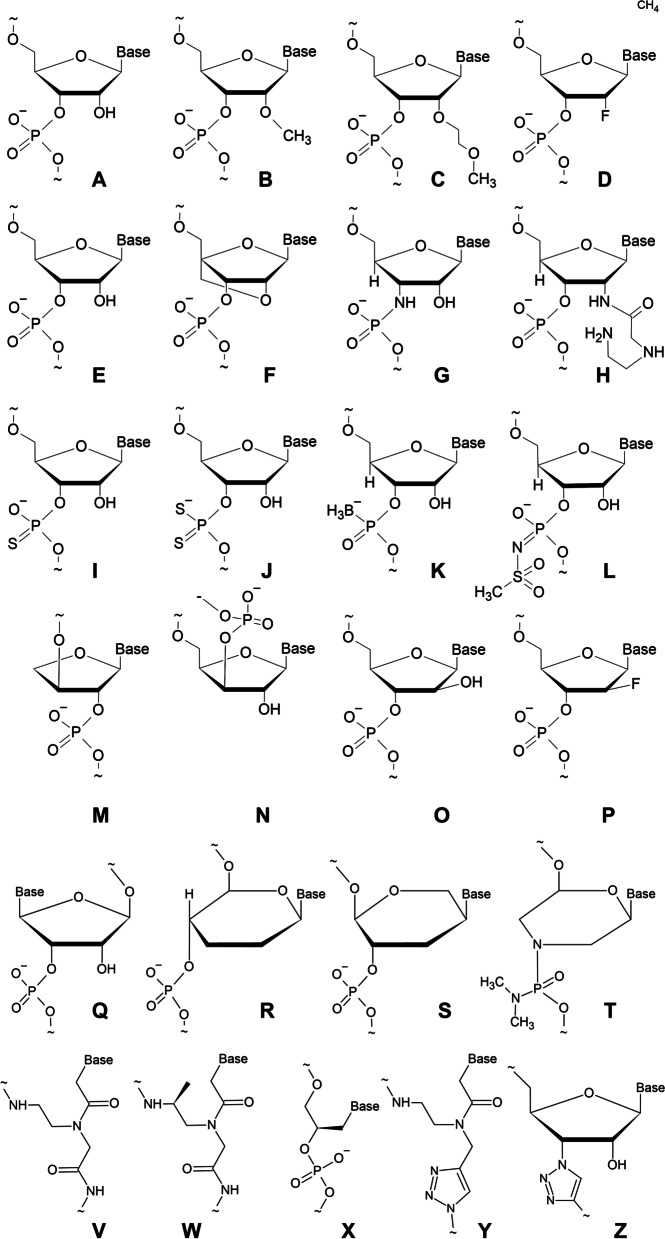
Structural formulae of AGPs with respect to native RNA (**A**). **B—**O-Methyl-; **C—**O-Methoxyethyl- (MOE); **D—**2’-deoxy-2’-fluoro- (2’-F); **F—**locked nucleic acid (LNA); **H**,—2′-O-(N-(aminoethyl)carbamoyl)methyl-; **I**,—phosphorothioate; **J—**phosphorodithioate; **L**—mesyl phosphoramidate; **M**—TNA; **N**—XyNA; **O**—arabino nucleic acid; **P**—FANA; **Q**—L-ribose; **R—**homoDNA; **S**—hexitol; **T**—morpholino; **V**—PNA; **W**—γ-PNA; **X—**GNA; **Y**—triazole-linked PNA; **Z**—triazole-linked RNA

Any ventures into the modifications of the structural backbones of biological polymers using alternative building blocks may be interesting for many reasons, but pose significant risks in terms of creating somethings completely non-functional. Many imaginable substitutions in the (deoxy) ribose phosphate backbone of natural nucleic acids have been tried but frequently yielded polymers with properties too different from natural nucleic acids. For example, homo-DNA (hexose instead of pentose, Fig. 1R) showed preponderance to avoid formation of double helices, strongly preferring linear duplexes, and generally exhibiting much weaker WC (Watson–Crick) bonding [15]. Also, neutralization of phosphates readily leads to AGPs that retain the ability to bind DNA/RNA only in a triplex-forming fashion [16].

The most obvious idea for modifications to the DNA/RNA backbone is to substitute sulfur (or even selenium) for oxygen in the phosphate groups (phosphorothioate**, **Fig. 1I, and phosphorodithioate, Fig. 1J). The phosphorothioate oligonucleotides (PSO) are much less sensitive to nucleases, and this feature allowed them to become therapeutically important. Sometimes PSOs also demonstrate much higher affinities to protein binders than native nucleic acids (reviewed in [17]). However, the phosphorus atom with sulfur instead of oxygen is a chiral center, therefore PSOs are composed of a mixture of a huge number of diastereomers. This fact, conversely, may indicate that currently used stereo-random PSOs may be greatly improved in the nearest future by recent breakthroughs in their chiral synthesis [18], although one cannot exclude a decrease in their biological activity if these mixed diastereomeres act in consort. Unfortunately, PSOs also exhibit a significant nonspecific toxicity in vivo [19]*.* For this reason, other modifications of the phosphate group may eventually replace phosphorothioates. For example, mesyl phosphoramidates (Fig. 1L) are superior to PSOs in some experimental settings [20], 20].

RNA is notoriously famous for its sensitivity to RNases due to the presence of 2’-hydroxyls. For this reason, various substitutions at this position have been tried, over a long period of time, and the simplest of them, 2’-O-methyl oligonucleotides, have found a great use in various applications (Fig. 1B). 2'-O-(2-Methoxyethyl)-RNA (MOE-RNA, Fig. 1C) are even more resistant to nucleases than PSOs [22], and this modification is widely used alone or in PSOs, however, some recently studied substitutions may become more potent, for example, 2′-O-(N-(aminoethyl)carbamoyl)methyl modification (Fig. 1H) holds promise to reduce the need for PSOs [23].

2’-Deoxy-2’-fluoro oligonucleotides (Fig. 1D)) are also widely employed, especially in aptamers. Moreover, it was 2’-deoxy-2’-fluoro-β-D-arabinonucleic acid (FANA, Fig. 1P) that was successfully used by DNA polymerases to synthesize not only AGP on a DNA template but, vice versa, DNA on AGP [24].

The next obvious idea to prepare nuclease-resistant oligonucleotides is to use their mirrors with L-ribose instead of normal D-ribose (Fig. 1Q). This was a technically challenging task until the breakthrough in 2016 which introduced the so-called spiegelmers: Wang et al.[25] reported on the transcription of L-oligonucleotides by a polymerase that was composed of chirally mirrored D-amino acids. Based on these achievements, the whole process became more straightforward, including the step of systematic evolution of ligands by exponential enrichment (SELEX) with libraries of DNA or RNA aptamers followed by their conversion to the corresponding spiegelmers that would bind the natural target.

One of the most successful AGPs is the Peptide (or Polyamide) Nucleic Acid (PNA), which was first synthesized and studied by Peter Nielsen et al. [26]. This PNA backbone was a linear polymer of N-(2-aminoethyl)glycine, where the amine group serves for attachment of nucleobases through a methylenecarbonyl linker (Fig. 1V). PNA-DNA hybrids were found to be more stable than corresponding DNA-DNA (higher melting temperatures). This was explained by the lack of “electrostatic repulsion” between strong negative charges of natural DNA. Moreover, the ability of the PNA to quickly displace one strand in the dsDNA has also been noted. From this early success the basic principles for the design of AGPs had been clearly formulated: the artificial analog must be homomorphous to native nucleic acids with respect to the number of backbone bonds (six per repetitive unit) and the distance between the backbone and nitrogenous bases. However, since homopyrimidine PNAs also showed a tendency to form triple complexes, it took a significant effort to demonstrate the ability of PNA to hybridize with complementary sequences of DNA, RNA, or PNA itself, according to the Watson–Crick hydrogen bonding rules [27]. Together with this, the authors described interesting properties of new polymers, for example, much greater thermal stability of PNA complexes that becomes equal to that of natural DNA only at a very high concentration of salt (i.e. ionic strength). Later, other amino acids were tried in PNA with various success, for example, phenylalanines may increase the mismatch discrimination of PNA upon hybridization [28].

Later, it was found that extending the PNA unit by an additional methylene had a strong negative effect on triplex stability, thus PNA is near-perfect for binding double-stranded nucleic acids in their A-form-like conformations [29].

The discovery of PNA instigated numerous studies on other similar AGPs. For example, the synthesis of pyrrolidine nucleic acids, bepPNAs, was described with the aim to reduce conformational flexibility of PNA [30], resulting in the preferential binding to RNA over DNA. On the contrary, pyrrolidinyl peptide nucleic acid carrying a D-aminopyrrolidine carboxylic acid demonstrated a significant preference to DNA over RNA [31]. Pyrrolidinyl PNA with (2'R,4'R)-proline/(1S,2S)-2-aminocyclopentanecarboxylic backbone (acpcPNA) also prefers DNA, and, moreover, its binding to RNA is highly sequence-specific [32, 33].

We should emphasize again the inherent flexibility of PNA which presumably exists in a disordered state, and that only binding to DNA/RNA fixes PNA in a defined conformation. Therefore, numerous attempts to introduce rigidifying groups have been attempted. For example, cyclopentane rings in one or multiple positions in PNAs strongly increase the melting temperature to complementary targets [34]. Introduction of cyclohexanyl PNAs (chPNAs) demonstrated that PNAs may bind RNAs in a highly preferential manner over DNA [35].

The morpholino oligonucleotides (MPO, Fig. 1T) with molecular structures based on methylene morpholine rings and phosphorodiamidate bonds were first developed by Summerton and Weller in 1991 [36]. Since then, PMOs have gained much support and use in antisense techniques to change gene expression, since, for example, in amphibians or fishes they work better than siRNA with less off-target effects.

Backbones less divergent from the natural DNA also attracted significant attention in terms of the systematic synthesis of all isomeric alternatives to the natural backbone, especially with respect to the analysis of alternative replicating nucleic acids and the origin of life. These new structures included various sugar isomers. For example, β-D-ribopyranosyl-(4′ → 2′)-oligonucleotides (p-RNA) showed strong and specific WC bonding [37]. Further studies on pentopyranosyl-(2'– > 4') oligonucleotides were found to form stronger complexes according to WC rules [38]. It is interesting that in the 2'– > 4' system, four out of eight possible isomers give efficient WC base paring, whereas in the 3'– > 5' system only two are possible (RNA and its arabino-isomer, although with a lower stability). These variants, however, were found orthogonal to natural NA, as they have not demonstrated pairing with DNA or RNA, thus decreasing interest in further studies.

Many other backbone systems were at first deemed to be incompatible with the retention of the useful properties of natural nucleic acids. However, it turned out that sometimes it is possible to depart from the strict rule (*six covalent bonds per repetitive backbone unit*): L-α-lyxopyranosyl (4‘ → 3‘) oligonucleotides also showed cooperative base-pairing [39]. This observation indicated that other variations may be permissive and resulted in a breakthrough discovery of TNA ((L)-α-threofuranosyl oligonucleotides (Fig. 1M) with (3'– > 2') phosphodiester bridges [40]. TNA is capable to base-pair according to WC rules not only with itself but also with DNA and RNA, although their thermal stabilities were widely different in homobasic and heterobasic combinations. Importantly, TNA was found to be more hydrolytically stable than RNA.

Methylene-extended TNA, phosphonomethylthreosyl nucleic acid (pTNA), a unique type of XNA with the C-P bond, does not base pair with DNA or RNA. However, historically, it was one of the first candidates for xeno-world. Indeed, directed evolution has yielded polymerases that can transcribe the DNA sequence information into pTNA [41, 42].

From 1997, a number of deeply modified nucleic acids, called locked or bridged nucleic acids, have successfully been synthesized after numerous efforts to prepare more and more stable analogs of RNA by modifying 2’-hydroxyl. Moving away from simple modifications like 2’-O-methylation, the Imanishi group has synthesized nucleosides where the methyl group was connected via methylene to C-4 of the ribose ring [10]. Independently from this study, in 1998, the Wengel group reported the synthesis of similar oligonucleotides [11]. Here we prefer the term “locked nucleic acids” (LNA) and would like to say that they turned out to become the most successful among all of the AGPs known to date.

The chemical nature of the key element in LNAs, the locking bridge between 2'- and 4'-positions in the furanose ring, may be different: via O-methylene (oxy-locked nucleic acid, LNA sensu stricto, Fig. 1F), S-methylene (thio-locked nucleic acid), or amino-methylene (amino-locked nucleic acid). They all can lock the ribose base in a conformation close to that of RNA or DNA in the A-form. More specifically, they can be fixed in one of the puckers, C3'-endo type (called β-D-LNA) or B-type conformation (C2'-endo). The former displays remarkable increases in melting temperatures. Like PNA, LNA oligonucleotides have an increased stability inside the cell and their hybrids with DNA and RNA have a much higher thermal stability in comparison with hybrids formed by natural oligonucleotides [43]. These features are extremely useful in antisense technologies for the suppression of gene expression and for identification of single nucleotide polymorphisms in various detection protocols. Depending on the task, the modified nucleotides (PNA, LNA, etc.) can be used not just in a uniformly modified format, but can also alternate with natural nucleotides in the same artificial polymer. The former are organized in blocks are called "Gapmers", whereas the chains with interchanging variants are denoted as "Mixmers".

LNA is a type of AGP with increased backbone rigidity. In such systems, bonding specificity should be increased. Indeed, the importance of these peculiarities may also be illustrated by a curious observation about the preference of homoDNA (Fig. 1R) to form duplexes enantio-selectively, but preferring strands of opposite chirality [44].

**Fig. 2:**
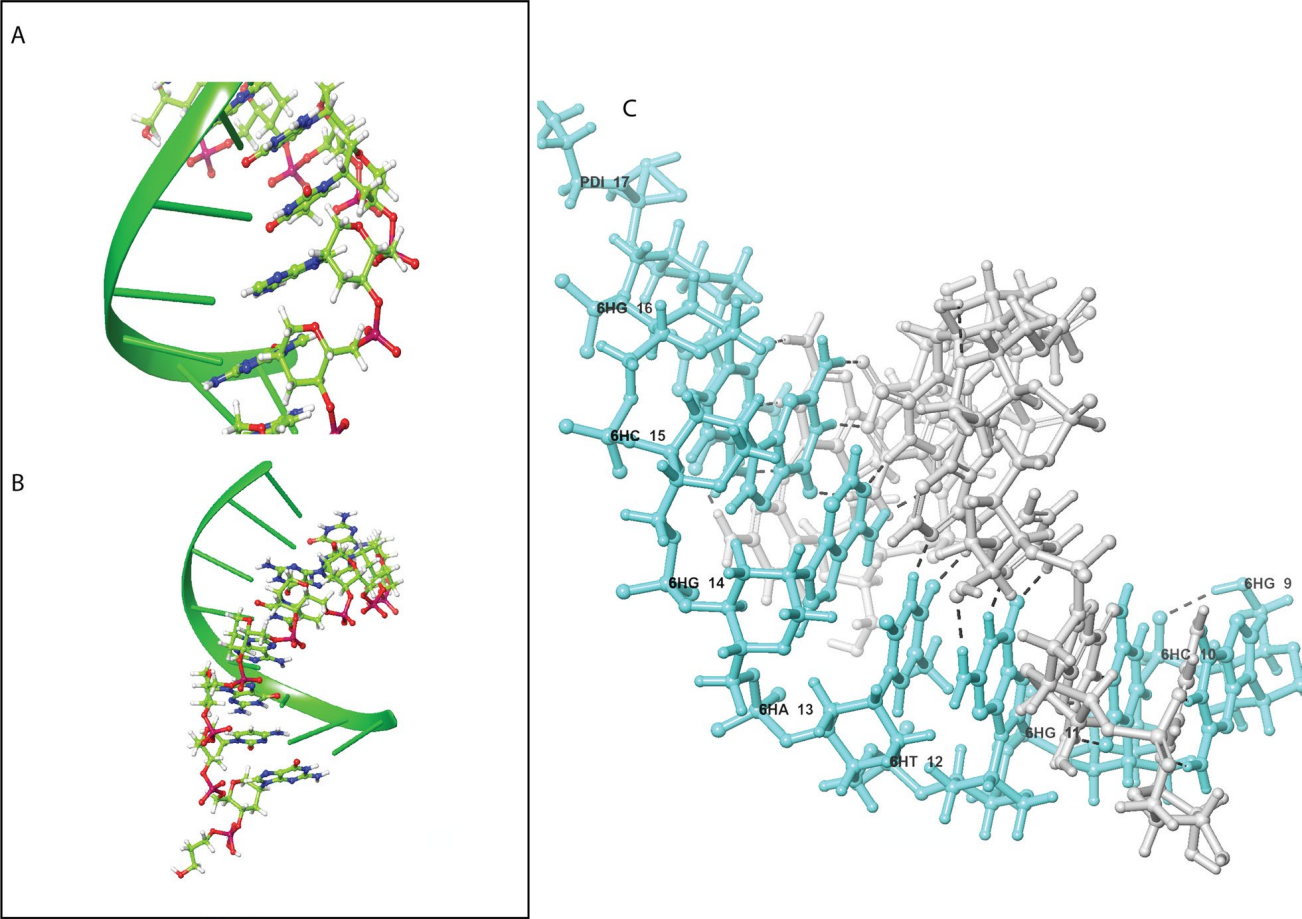
Example of AGP-RNA complex three-dimensional structure, HNA: RNA complex. **A:** Precise orientation of HNA (in skeleton scheme) wound around the single-stranded RNA structure (green cartooned color); **B:** Representation of HNA interaction with RNA backbone; **C:** This shows the hydrogen bonding between RNA (in white) and HNA (in cyan). The HNA is labeled according to the scheme (h-base-α in the structure where h = anhydrohexitol, Base = AGTC nitrogenous base, α = position of residues in the complex structure). PDBID:1EJZ [47]

In contrast to the rigid LNA, a maximally flexible Unlocked (UNA) was synthesized a long time ago (reviewed in [45]). Variations of this theme give other acyclic AGPs that should be termed true “xeno” nucleic acids: GNA, FNA etc. The glycerol nucleic acid may be either (S)-GNA and (R)-GNA (Fig. 1X) and their strands self-assemble into homo-chiral antiparallel right-handed and left-handed helices [46] (Fig. 2).

**Fig. 3:**
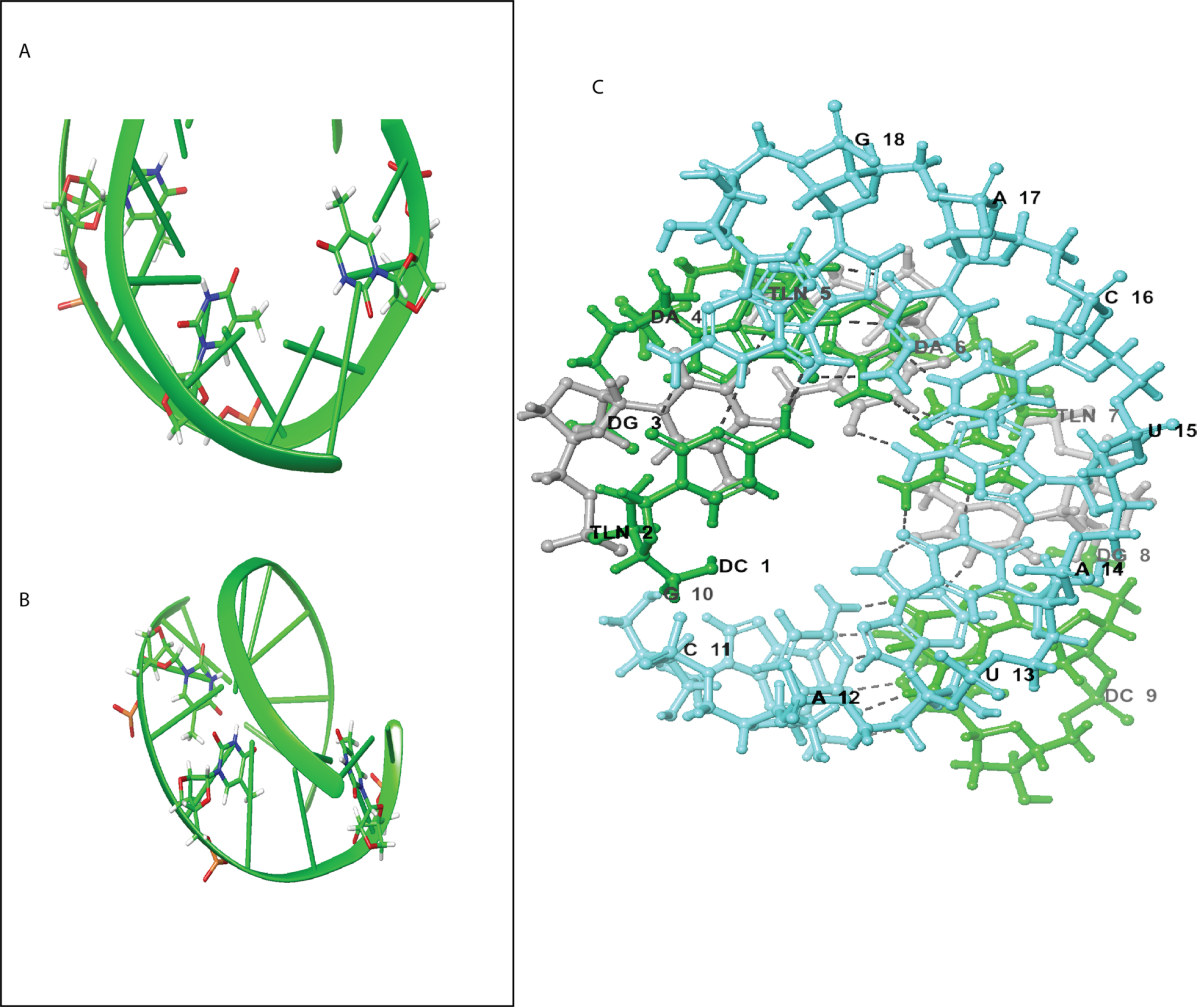
Example of AGP-RNA complex three-dimensional structure, LNA:DNA:RNA complex. **A:** Cartooned representation of the complex structure, wound around the RNA strand (green cartooned color); **B:** Full representation of interaction with RNA:LNA: DNA backbone; **C:** Hydrogen bonding between DNA-modified (green), LNA (white), and RNA(cyan). The scheme used in naming the structure is denoted as Dα, where D = DNA and α = nucleotide base; TLN = thymine LNA nucleotide. The numbers are according to the position of respective residues in the naming scheme. Additionally, ATCG represents the standard nitrogenous bases present in the structure. PDBID:1HHX [49]

**Fig. 4:**
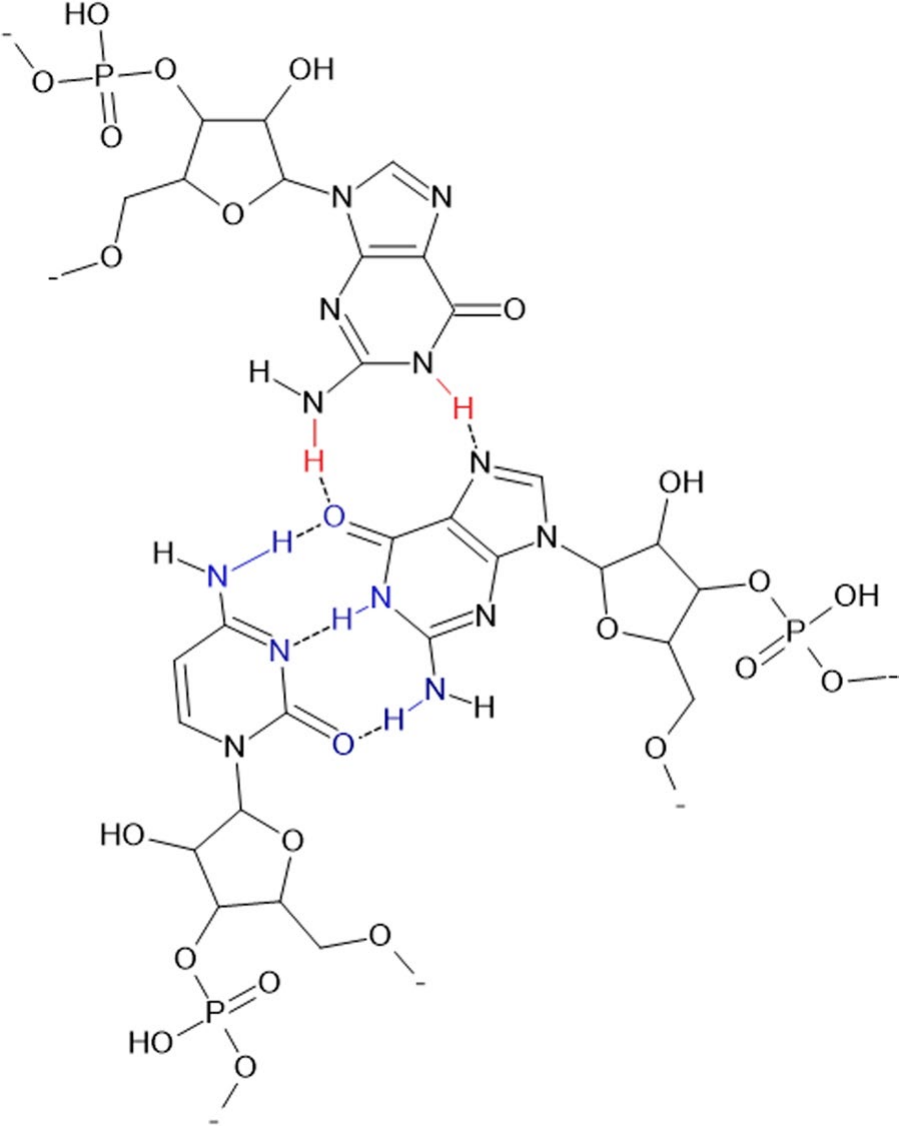
Watson–Crick (WC) and Hoogstenian (HG) base pairing. GGC triplex is shown. Note that in schemes WC interaction (participating bonds and atoms shown in blue) is usually denoted by "-" and the HG interaction (shown in red) by " * "

Finally, linkages with very different chemistries should be exemplified the best by triazole-linked AGPs since they are produced by the established click synthesis, such as the analog of PNA in Fig. 1Y and the analog of RNA in Fig. 1Z (reviewed by Baraniak and Boryski [48]). Properties of some AGPs with respect to native nucleic acids are summarized in Table 1, examples of solution 3D structures of AGPs complexed with native nucleic acids are given in Figs. 3 and 4

**Table 1:** Effects of backbone modifications on some functional properties of nucleic acids

Modification	Duplex stability	Hybridization specificity	Sensitivity to pH changes	Sensitivity to ionic strength	Solubility	RNAse resistance	RNAse H activation
PNA	↓	↑	↓	↓	↓	↑	↓
LNA	↑	↑	↓	↓	↓	↑	↓
PSO	↓	N/A	N/A	N/A	↓	↑	N/A
MOE, 2’-O-Me	↑	N/A	N/A	N/A	↑	↑	N/A
2’-F,2’-deoxy	↑	N/A	N/A	N/A	↑	↑	N/A
Triazole-linked	↓	N/A	N/A	N/A	N/A	↑	N/A

↑ and ↓ indicate increase or decrease with respect to unmodified DNA or RNA, reviewed in more detail in [70]

**Table 2:** Oligonucleotide drugs approved for therapeutical use

Drug / Trade name	Mode of action	Backbone / Modifications	Disease	Target	Route
Viltolarse /Viltepso	ASO	PMO	DMD	Dystrophin, exon 53	i/v
Fomivirsen / Vitravene	ASO	PSO	Cytomegalovirus retinitis	Cytomegalovirus infection	i/vt
Mipomersen / Kynamro	ASO	PSO, MOE	Homozygous familial hypercholesterolemia	APO B-100	s/c
Iotersen / Tegsedi	ASO	PSO, MOE	hATTR amyloidosis-polyneuropathy	Transthyretin	s/c
Eteplirsen / Exondys 51	ASO	PMO	DMD	Dystrophin, exon 51	i/v
Golodirsen / Vyondys 53	ASO	PMO	DMD	Dystrophin, exon 53	i/v
Nusinersen / Spinraza	ASO	PSO, MOE	Spinal muscular dystrophy	SMN2	i/t
Patisiran / Onpattro	siRNA	2’-O-Me	hATTR amyloidosis-polyneuropathy	Transthyretin	i/v
Pegaptanib / Macugen*	Aptamer	2’-O-Me, 2’-F, PEG	Macular degeneration	VEGF	i/vt
Lumasiran / Oxlumo	siRNA	2’-O-Me, 2’-F, PSO	Primary Hyperoxaluria type I	Hydroxy Acid oxidase	s/c
Casimersen / Amondys 45	ASO	PMO	DMD	Dystrophin, exon 45	i/v
Defibrotide / Defitelio	DNA	ssDNA from pig intestines	Hepatic Veno-Occlusive disease	multiple mechanisms	i/v
Inclisiran / Leqvio	siRNA	2’-O-Me, 2’-F, PSO, trGalNAc	Familial Hypercholesterolemia	PCSK9	i/m
Volanesorsen / Waylivra**	ASO	PSO, MOE	Familial Chylomicronemia	Apolipoprotein C-III	s/c
Givosiran / Givlaari	siRNA	2’-O-Me, 2’-F, trGalNAc	Acute Hepatic Porphyria	5’-aminolevulinic acid synthase 1	s/c

*ASO* Antisense Oligonucleotide, *i/v* intravenous, *i/vt* intravitreous, *s/c* subcutaneous, *i/m* intramuscular, *i/th* intrathecal, *MOE* 2’-O-methoxyethyl, *2’-FP* 2’-deoxy-2’-fluoro, *PSO* Phosphorothioate, *PMO* Phosphorodiamidate morpholino, *trGalNAc* triantennary N-acetylgalactosamine, * discontinued, ** approved in Europe, *DMD* Duchenne Muscular Dystrophy, *hATTR* Hereditary Transthyretin, *PCSK9* Proprotein Convertase Subtilisin/Kexin type-9

### Binding of AGPs to DNA as anti-sense oligonucleotides (ASO) or triplex-forming oligonucleotides (TFOs)

In order to target double stranded nucleic acids in a complementarity dependent manner, it is necessary to displace one strand of the target. However, there are serious difficulties in the use of flexible AGPs like PNA for duplex invasion. Therefore, Bis-PNA consisting of two functional blocks connected by a linker represents a very attractive approach: bis-PNAs with two PNA strands and a linker such as 8-amino-3,6-dioxaoctanoic acid were a much better tool for targeting the double-stranded DNA via strand invasion than monomeric PNAs [48]. Virtually the same ideology as for bis-PNAs can be applied for the use of bis-LNAs. Their successful development and application for invasion into chromosomal DNA was reported in the works of Moreno et al. [50] and Geny et al. [51].

The idea of employing triplex formation instead of invasion into the preexisting duplex [52] gained much support and attention—TFO (triplex-forming oligonucleotides) bind the major groove by Hoogsteen (HG) pairing, although the triple helix is believed to be less stable than the double helix [53]. Indeed, the HG discovery by Christ Hoogsteen [54] are important for the formation of triplexes. This is sometimes called "non-canonical" bonding. However, this is just another natural way of interaction between the nucleotides in the complementary nucleic acid chains. In HG bonding two antiparallel nucleic chains form hydrogen bonds only along the large groove (Fig. 4), whereas two DNA helices are in the anti-parallel orientation with the "canonic" WC interaction and a third strand of DNA connected to one strand by the HG interaction. Essentially, it should be emphasized again that the HG mechanism efficiently works in the formation of triple helix nucleic acids [55].

A homopyrimidine PNA may easily target homopurine DNA via the creation of the so-called P-loop formation [12], where the non-complementary DNA strand is left in a single-stranded state. Interaction of PNA with dsDNA is sensitive to pH, and may be adjusted by the inclusion of intercalators into the reaction mix [52]. However, the sequence-specific targeting by AGP TFOs is more challenging in comparison to strand invasion. For these purposes, other techniques seem beneficial, for example, the PD-loop mechanism that requires two PNA "openers", and one ssDNA binding oligonucleotide [56]. In the “tail-clamp” approach, a PNA consisting of a short (e.g., hexamer) sequence forms a triple helix and another sequence forms the required duplex with the second strand of the target DNA according to WC rules [57]**.**

PNAs consisting of a homologous hybridization site, a hinge, and a polypurine site forming a triplex—reverse-Hoogsteen mating with the first site chain is known as "polypurine reverse-Hoogsteen" /Polypurine reverse-Hoogsteen (PPRH). These structures form a triple helix or a guanine quadruplex (G4), which proved to be efficient in knocking down gene expression [49, 58]. Interestingly, the most effective TFOs may be created by alternating mixmers of LNA and native DNA, since they may pre-set the hetero duplex towards the triple-helical-like conformation [59]. Also, G-quadruplexes can be targeted with PNA [60]. There is a low dependence in DNA-PNA hybrids on the ionic strength for thermal stability. In contrast, the PNA backbone has a neutral charge [23]. The second feature is the flexibility (or even softness) of the PNA molecule, capable of adjusting to its partner’s shape. In 1996 it was [61] demonstrated that the anti-parallel complex of PNA:DNA may have a unique form, which displays features of both the "B" and "A"-form of the double helix of nucleic acids. Similarly, it was established that the anti-parallel duplex PNA:RNA closely resembles the A-form, which is logical since RNA usually dominates in the RNA:DNA duplexes [62, 63]. The third feature of the interaction of AGP-like PNAs with DNA is an enhanced selectivity of binding compared to that of natural nucleic acids—in other words, mismatches between PNA and DNA are more destabilizing than those in DNA/DNA duplexes, as shown by Demidov et al. [64]. The fourth feature is the lower solubility of the unmodified PNA. And, most importantly, the solubility of PNA decreases with increasing polymer length, as the degree of aggregation increases. Hence, the synthesis of long molecules, especially rich in purines, is problematic [65] to the DNA-DNA duplexes, where the increasing ionic strength of the solvent shields the repulsion of negatively charged phosphates.

In order to understand the properties of the triple helices with AGPs, it is necessary to briefly review the conditions of triple helix stacking of canonical nucleic acids. First, triple helixes can be either parallel or anti-parallel. This is determined by whether or not the direction of the third helix from the 3' to the 5' end coincides with the first helix, with which it has the aforementioned HG interactions. In triple helixes, the third chain is located in the large groove of the double helix duplex formed by the first two chains. The parallel triple helix can include three nucleoside triplets: T*A-T, C + *G-C, and G*G-C, each stabilized by two HG bonds. The anti-parallel triplet can include four triplets: T*A-T, A*A-T, G*G-C [15, 24], and C + *G-C. The stability of chains of the "C + *G-C" kind requires protonation of cytosine in the third chain for the formation of Hoogsteenian hydrogen bonds, therefore, the stability of this kind of triple helix strongly depends on the acidity of the medium: the helix is stable at pH 5 and loses the third strand (DNA) at pH 7 or higher. It should be noted that the stability of such a triple helix, called H-form DNA, depends not only on the pH of the medium, but also on the torsional stress of the DNA strand. These effects were investigated in detail in the Frank-Kamenetsky laboratory [66].

Antiparallel triplets are characterized by their sensitivity to the presence of metal salts. Stabilization of triple helixes with MgCl_2_, and the blocking of the third helix binding by 100 mM KCl was noted by Cheng et al. [67]. However, in the case of AGPs these factors remain less studied, and many accessory approaches may be employed therefore. For example, pseudocytosine was substituted for cytosine in the PNA region that forms the third chain [68]. The resulting bis-PNAs form three-chain complexes with higher thermal stability, and the pseudocytosine decreases sensitivity to pH.

An interesting approach was used in the work of Glaser's group in 2007 [69]: to increase binding specificity, a pair of pseudo-complementary PNAs was used, capable of binding to the forward and reverse strands of genomic DNA, but unable to form a stable bond with each other due to replacement of adenine and thymine with 2,6-diaminopurine and 6-thiouracil, which are destabilized during hybridization together due to the steric interference. This was announced as a new tool capable of solving the problem of targeting (as mentioned above, the triple helix is not formed on all sequences, but only on oligo-purines or oligo-pyrimidines). An equally interesting, but  more straightforward approach to increase binding specificity was published by Demidov et al. [70] who proposed PNA-assisted affinity capture (OPAC) that combines two PNA probes as openers in the PD-loop to displace one of the target DNA chains, 20–30 nucleotides long. In addition, the probe is designed to be complementary to the displaced strand and carry a label of choice [69]. This approach was later used for native (without denaturation) labeling of specific DNA sequences [71], which essentially refers to the FISH variants discussed below.

## In vitro labeling applications

### *PNAs and LNAs demonstrated many successful applications *in vitro

1. PCR: blocking by specific binding to undesirable templates. A sequence-specific PNA clamps can be used, for example, for simultaneous detection of many templates or to create an expression library. It is possible to prepare such a set of PNAs that would block the amplification of undesirable cDNAs, favoring rare variants.

A new method of differential staining of different real-time PCR products was proposed by Ahn et al. [72] using PNA molecular beacons (oligonucleotides with self-complementary termini equipped with a fluorophore and a fluorescence quencher on the other end, allowing a great increase in signal intensity upon hybridization to a PCR product being synthesized). Authors used PNA beacons for successful simultaneous detection of three mycoplasma species in the same tube via fluorescence melting curve analysis (FMCA), yielding higher melting temperature and specificities in the PNA-DNA hybrids. A 2019 paper by Jeon et al. [73] compared the detection method of EGFR mutations in patients with lung cancer using the "classic" PNAClamp method with a new approach combining PNAClamp and PAGP S-Melting (PAGPMutyper™). First, real-time PCR blocks the wild-type PCR product due to the strong interaction of the fixing PNA (PNAClamp) with the wild-type template, and if a mutation is present at this site, the fixing PNA binding is not strong enough to block the PCR reaction. In the next step, the PCR products containing the mutations bind to specific PNA molecular beacons with different fluorophores and quenchers that correspond to the sequences of different mutant sites. Since the melting temperature of PCR products with the corresponding PNA depends on the sequence, different products have different melting temperatures, which makes it possible to reliably detect several mutations of a particular gene in a single tube.

2. Capturing of targets for various sensors and microarrays

Detection of mutations or allelic variants using microarrays benefits from the sensitivity of PNA to nucleotide mismatches in complementary nucleic acid, which is higher than that of DNA [74]. In this respect, Choy et al. [75] compared single-nucleotide polymorphism microarrays based on PNA and DNA. According to the researchers' estimates, PNA-based microarrays were 2.0–37.3 times more specific and approximately 10 times more sensitive than DNA-based microarrays. Earlier, a paper was published that formulated a series of rules for creating mismatch-sensitive nucleotides synthesized from conventional and locked nucleic acids by You et al. [76]. Embedding with a single LNA monomer in the melting probe allows differentiation of four SNP alleles by four distinct melting temperatures thanks to different effects of the LNA substitution on mismatches [77]. However, it should be noted that despite their high affinity to DNA and RNA, high selectivity, and enzymatic stability, PNA has not yet displaced conventional DNA oligonucleotides in microarray techniques with the exception of detection of allelic polymorphisms.

3. Fluorophore-labeled probes.

The advent of PNA has significantly improved the hybridization process due to its higher affinity for natural nucleic acids. In 2005 the work by Agerholm et al. [78] was published, where probes specific to different chromosomes were prepared. When comparing the PNA-based probes with conventional probes, it was shown that PNA-based probes can withstand a large number of rehybridization cycles, which makes it possible to perform several stainings for different chromosomes on one sample. This is very important in the work on preimplantation genetic diagnosis for screening embryos for aneuploidy.

An interesting approach is described in Giunta and Funabiki [79] to investigate the maintenance of human centromere integrity by CO-FISH (chromosome orientation-FISH). The use of DNA primers increases the resolving power of this method and makes it possible to detect changes in the block structure of higher order repeat (HOR) DNA sequences that make up centromeres. The work demonstrated the disturbance of centromeres' structure during cell aging and cancer.

It should be noted that technological advances have now come to the possibility of working with single molecules. Application of these approaches to study the polymorphism of individual genes and separate labeling of paternal and maternal chromosomes can be seen, for example, in the work of Bryan and colleagues [80].

The use of PNA probes for diagnosing parasitic infections has a long history. For example, probes for the identification of tuberculosis mycobacteria were created back in 1999 [81] and this direction has been successfully developed since then. In 2006, a paper by Lefmann et al. was published, presenting the data on the increase in the number of strains that can be successfully detected with the use of PNA [82].

Probes based on AGPs are also used for the diagnosis of fungal infections. The high affinity of DNA mimetic probes has helped to increase the reproducibility of measurements to a level that meets the requirements of the US Food and Drug Administration (FDA) and the European Medicines Agency (EMEA). In spite of the higher cost (somewhere between 40 and 200-fold) of the PNA samples, compared to DNA oligonucleotides, the savings made per patient was about $1,800 when testing his blood for *Candida albicans*, a common pathogenic flora of hospitals. The *in-situ* hybridization method allows the use of standard formaldehyde-fixed and paraffin-embedded histological sections for analysis with an antisense PNA probe targeting a unique Trichosporon 26S rRNA sequence, and the probe for the detection of *Candida albicans* [83].

Moreover, LNA primers work better, for example, even in Sanger sequencing [84]. Interestingly, unlocked nucleotides may also be useful in PCR primers for allele-specific analysis [77]. 2'-amino-LNA are especially interesting since the 2'-amino group can be used for the attachment of fluorophores and other labels [85]. Even in isothermic amplification protocols the use of PNA may be of value [86]. For a broader coverage of AGPs application in diagnostics an excellent review [87] can be recommended.

4. Self-assembly of supramolecular structures.

For example, bis-PNA has been used to bring two molecules together to create self-assembled DNA nanostructures, and this may have implications for the future design of DNA computers, and other nano-devices [88, 89]. For example, *g*amma-modified PNAs (γPNA, Fig. 1W) can form self-assembling bundles of nanofibers in some organic solvents [90]. PNA and other AGPs may assist in the creation of pharmacological materials with predetermined properties. For example, a film material consisting of paired layers of PNA resistant to proteosomal and nuclease degradation may be doped with DNA to obtain materials with precise non-zero degradation rate of in the organism [91].

5. Probing the chromatin structure and other protein-DNA/RNA interactions.

Investigation of various DNA/RNA binding proteins may greatly benefit from more stable hybridization of AGPs to complementary targets and changes in protein-binding modalities (for example, [92]).

DNA mimetics also provide a versatile toolkit for chromatin research. For example, Brown et al. [93] described the creation and application of a "simple" in vitro chromatin system simulating the epigenetic features of various posttranslational modifications of histones. In brief, phased nucleosomes of "pure", unmodified histones were assembled on DNA. Then, a pre-modified peptide representing the N-end of histone H3 with methylated lysine at the H3K27 position was attached to the PNA designed to bind the DNA sequence in the vicinity of the N-terminus of nucleosomal histone H3. This artificial mixture mimics the N-terminal portion’s covalently modified histones. The authors then compared the methyltransferase activity of PRC2 on the modified and control substrate to test the hypothesis of H3K27 methylation spreading across chromatin from one "seed" site.

In the work of Boffa et al. [94] a biotin-labeled PNA was used to isolate chromatin enriched with a tandem CAG repeat typical for several inherited neurodegenerative diseases, caused by expression of a mutant protein characterized by extensive poly-lysine sites.

Binding of a hairpin polyamide dimer only to a DNA sequence located on the crustal part of the nucleosome and phased in a certain way was described in Edayathumangalam et al. [95]. One part of this construct is located in the NA groove of the first DNA strand wrapped around the nucleosome, and the remaining part is located in the groove of the other strand. The authors significantly advanced in solving the problem of specificity of binding to DNA within the nucleosome. The DNA on the surface of the nucleosome is accessible at best seven nucleotides in a row. This stretch is followed by 3 nucleotides of the ten-nucleotide DNA helix, pressed to the body of the nucleosome. Given the fact that seven nucleotides can be repeated tens of thousands of times in the human genome, this makes this approach not very favorable for the targeted binding. On the other hand, the binding of PNA to natural DNA within nucleosomes revealed that histone tails can prevent the PNA-DNA association [96], while the DNA region in contact with the nucleosome, on the opposite, can participate in the binding. This feature of PNAs raises an interesting possibility of using PNAs for genome editing purposes (discussed below).

## In cellulo applications

AGPs find very diverse applications in cell research. Ryo and colleagues in 2013 [97] developed a biosensor to measure microRNA levels in living cells. MicroRNAs are short (~ 22 nt) double-stranded non-coding RNAs that target the corresponding mRNAs for degradation. Not surprisingly, microRNAs regulate various important processes both in normal and diseased cells [98, 99]. An additional level of regulatory complexity is introduced by the fact that microRNAs can be sponged by long non-coding RNAs. The latter are often derived from anti-sense RNAs [100, 101].

Elmén et al. (2008) successfully used LNA that was complementary to the microRNA miR-122 seed site to reduce its expression in primates. The working concentration of miR-122-targeting LNA was in the range of 10 mg kg^−1^ [102].

Applications for cultured cells can also be divided into classes based on the target and mechanism of action. The targets can be primarily RNA (single- and double-stranded, either ASO or siRNA), DNA (also single- and double-stranded), and DNA/RNA-binding proteins. This oligonucleotide targeting mode of action may be mediated by ASOs, siRNAs, TFOs, aptamers, RNazymes/DNAzymes, etc. For example, the work by Musumeci et al. [103] chose the HMGB1 protein as a target. It is an intranuclear chromatin-binding protein involved in DNA repair that increases the mobility of nucleosomes thereby improving the binding of a number of sequence-specific factors. On the other hand, when entering the extracellular space, it displays the properties of a late cytokine involved in the onset of inflammatory diseases. Musumeci et al. [103] created a curved PNA/DNA duplex that binds avidly to HMGB1. In a mouse model of generalized sepsis induced by the administration of bacterial lipopolysaccharide, the results were a significant reduction in mortality.

A number of studies have attempted to partially disable the NF-κB signaling pathway that plays an important role in the immune response. For example, Mishatiati et al. [104] explored the properties of a monomer of the palindromic sequence in the NF-B binding site of the p52 subunit in the human immunodeficiency virus genome. As a result, they succeeded in obtaining the NF-κB p52 binding to the DNA-PNA heteropolymer, and, moreover, the binding was less stable than for the DNA-DNA sequence. Further, in 2002, a chimeric double-helix nucleotide has been proposed, a PNA-DNA-PNA (PDP) structure. It also showed the ability of PDP polymers to bind liposomes for decoy delivery into the cell [105]. In 2012, Finotti et al. [106] showed a striking reduction in IL-8 expression when a chimeric double-helix polymer was added to cells. Simultaneously, chromatin immunoprecipitation (ChIP) showed a decrease in the number of NF- κB molecules at the IL-8 promoter. This approach looks promising in the treatment of various genetic diseases, for example, cystic fibrosis.

Similarly, attempts were made to deplete cells of the transcription factor Sp1, which contributes to an increased expression of urokinase type plasminogen activator receptor (μPAR), that in turn strongly correlated with the metastatic potential of melanoma, breast, lung and colorectal cancer cells. It was shown that the best binding is ensured by chimeric (DNA/PNA) molecules [107, 108] consisting of a central chain of DNA, containing the Sp1-binding sequence, flanked by two PNA fragments annealed with the complementary DNA sequence.

### AGPs for intracellular degradation of mRNA

The effect of LNA was studied not only in vitro, or in cell lines, but also in vivo. As an example, in the work of Wahlestedt et al. [109] 15-nucleotide phosphodiester gapmers and mixmers injected into the rat brain were shown to suppress the expression of delta opioid receptors. A year earlier, in the work by Shammas et al. [110] the blocking of telomerase RNA by specific PNAs was described, resulting in the subsequent telomere length shortening and cell division arrest. The IC50 was 10 nM for cell extracts and 70 nM for permeabilized cells.

Hypoxia-inducible factor 1 (HIF-1) is a transcription factor ensuring cell and tissue survival in oxygen-deficient conditions. Primarily, its activation leads to increased expression of vascular endothelial growth factor (VEGF) and its receptors. This factor has been shown to play a critical role in angiogenesis in cell survival, metastasis, drug resistance, and glucose metabolism. Increased expression of HIF-1 α subunit (HIF-1α), which occurs in response to hypoxia or activation by growth factors, is associated with poor prognosis in many types of cancer. Greenberger et al. [111] described the synthesis and use of a gapmer sequence 5’-TGGcaagcatccTGTa-3’ (capital letters stand for LNA, and small letters for regular DNA), as an antisense for HIF-1 α. A significant decrease of the mRNA and protein levels upon the introduction of this LNA was observed in cancer cell lines. As a consequence, a decrease in the secretion of VEGF (Vascular endothelial growth factor) and MMP2 (matrix metalloproteinase-2) was also detected. Suppressive effects on cancer cells were also obtained in vivo, using a xenograft experiment—when thymectomized mice were injected with human prostate cancer cells.

Unfortunately, LNA oligonucleotides induce significant levels of hepatotoxicity [112], supposedly through the p53 and NRF2 stress pathways [113].

### Anti-protozoal, anti-bacterial, and anti-viral action of AGPs

The ability of certain PNAs to inhibit bacterial growth was shown shortly after the invention of AGPs. Thus, in the works of Good and Nielsen et al. [114, 115] the anti-sense PNA against the β-lactamase gene inhibited resistance to a wide range of β-lactam antibiotics in *E. coli* cells. Recently, numerous attempts have been made to block the bacterial chaperone dnaK in bacterial strains with multiple drug resistance. In this respect, an effect comparable to that of antibiotics was achieved with some strains of Salmonella [116].

When malaria was treated with low-molecular-weight drugs, drug resistance quickly emerged. The work of Kolezon et al. [117] described PNA -(dK)8TGGATAGT(TO)CCTTCTAG, where (TO) denotes the fluorescent dye triazole orange inserted in the middle of the molecule instead of adenosine, which decreased the expression of the PfSec13 gene encoding the bark protein of the nuclear pore complex in *P. falciparum*.

A more sophisticated approach was taken by Amit-Abraham et al. [118] on PNA against a large non-coding RNA responsible for switching the expression of surface antigens of malaria *Plasmodium falciparum* [118]. The plasmodia lost the epigenetic memory of the previously expressed VAR family antigens as well as a significant portion of its virulence due to the changing composition of the surface antigens.

In terms of numerous attempts to improve the delivery of AGPs into bacterial cells, one study has shown promise where diaminobutanoic acid (DAB) dendrons coupled to antisense PNA (anti-*acpP*) demonstrated a low toxicity for cultured human cells simultaneously with good bactericidal activity against *E. coli* and *Klebsiella pneumoniae* [119].

A suitable approach against viral infections was reported by Kesy et al. [120]: a PNA has been developed that efficiently binds to the double-stranded RNA in a "panhandle" structure. Notably, the influenza A virus carries eight single-stranded minus RNA chains. For a successful infection, firstly, mRNAs must be synthesized with capped primers. Secondly, the RNA must be replicated to form a positive RNA chain. To implement these processes, the virus uses a region of RNA with a complex three-dimensional switchable structure which also includes the previously mentioned "frying pan handle" type structure. By forming a triple helix RNA-RNA-PNA with a "panhandle," this PNA at a concentration of about 4 µM in the extracellular medium is able to reduce the amount of viral RNA to 30% of the control. To facilitate cellular uptake, the N-terminal uptake of PNA was conjugated to an aminosaccharide. It should be noted that the PNA sequence is slightly different from the classical "third chain." Modified bases forming additional hydrogen bonds were used for efficient binding: thio-pseudocytosine was used to bind to G-C, and guanidine-modified 5-methylcytosine was used for C-G pairs. The work also showed that the use of DNA complementary to the RNA chains did not demonstrate effective binding or a significant decrease in the titer of the influenza virus.

### Correction of splicing

The idea of controlling splicing appeared soon after the discovery of the mechanisms of its regulation. As early as in 1993 Dominski and Kole made the first successful attempt to restore the functionality of the mutated human β-globin gene (such mutations underlie the β-thalassemia disease) by changing its splicing [121]. For this purpose, modified antisense oligonucleotides, 2'-O-methyl-ribooligonucleotides against the mutated site, were used. It should be noted that this experiment was done in vitro on a nuclear extract and hence requires further validation in vivo.

A similar approach was used in the work of Dunkley et al. [122] to treat the dystrophin protein deficiency that causes Duchenne myodystrophy. This genetic disease is usually caused by mutations resulting in a frameshift and premature transcription termination. There is a milder variant of myodystrophy, Becker myodystrophy, in which the mutations do not cause frameshift and the mutant protein is present in the membranes of muscle cells. The work was performed on muscle cells derived from a mouse model of Duchenne myodystrophy in which a mutation in exon 22 of the dystrophin forms a stop codon. As the structure of the 2-O-methyl-oligoribonucleotides hybridized with RNA differs from the "natural" double-helix RNA this complex is not subjected to degradation by RNase H, which recognizes double-stranded RNA. As a result, during the splicing exon 22 is released as an intron and a functional mutant protein is obtained. The authors suggested that it may be possible to change the symptomatology of Duchenne myodystrophy (a severe prognosis) to the milder symptoms of Becker myodystrophy. Moreover, LNA-containing nucleotides were superior when compared to the corresponding 2'-O-methyl variants for dystrophin exon 23 skipping.

A similar approach was used in the work of Karras et al. [123]. There is an opinion that in some severe forms of asthma the amount of IL-5 receptor (interleukin-5) on the cell membrane plays an essential role. Thus, the authors investigated the possibility of reducing the amount of this receptor by switching off exon 9 in the mature RNA. This exon encodes the only transmembrane domain of the interleukin-5 receptor and without it a soluble form of the protein is produced that can compete for binding with interleukin-5, further reducing cell signaling. The work was performed on murine B-cell lymphoma BCL1. A set of 2'-O-methoxyethyl-modified ASOs overlapping the entire exon sequence plus four nucleotides of the previous and subsequent introns were used.

The work by Shiraishi et al. [124] studied the effect of PNA on stabilization of the tumor suppressor protein p53 in JAR cells (human choriocarcinoma) via disruption of splicing of its major negative regulator-ubiquitin ligase MDM-2. This was achieved by using PNA cross-linked via ethylene–glycol linker with the 9-aminoacridine ligand. Being the principal E3 ligase for p53, MDM2 binds the latter in the N-terminus causing ubiquitinylation of the C-terminus and subsequent degradation of the p53 protein [125–127]. Once stabilized, p53 exerts its tumor suppressive functions as a transcription factor affecting the expression of genes responsible for cell cycle progression, apoptosis, autophagy and metabolism [128–130]. The most successful PNA2512 molecule with the sequence: H-Acr-eg1-TTT GGT CTA ACC TAT -NH2 hybridizes with the MDM2 pre-mRNA at the intron 3/exon 4 junction. Of the 15 bases included in PNA, 4 come from the intron and 11 from the exon. PNA used together with capmtothecin (a Topoisomerase I inhibitor that causes its cross-linking to the DNA at the contact site) reduced the LD50 dose of capmtothecin by at least one order of magnitude.

Disruption of splicing in the PTEN gene, an important tumor suppressive phosphatase, by PNA was published by Wancewicz et al. [131] as a simple way to evaluate semi-quantitatively the efficiency of anti-PTEN PNA cross-linked with peptides for targeted delivery to various tissues.

In 2007 Beane et al. [132] compared the effect of a number of oligonucleotides against various regulatory elements in the promoter regions of the androgen and progesterone receptor genes in breast cancer cells. It was shown that transcriptional blocking was achieved specifically with LNA-based oligonucleotides, but not with 2'-methoxyethyl RNA (2'-MOE). The authors explain this by the fact that LNA forms stronger complementary bonds with DNA than 2'-MOE RNA. This was evident from the lower melting temperature of MOE-DNA heterocomplexes compared to the LNA-containing ones. It was also found that gene expression is most efficiently blocked by oligonucleotides that bind DNA in the vicinity of the transcription start site rather than to the binding sites of transcription factors. A certain exception to this rule was demonstrated for the Sp1 transcription binding site LNA oligonucleotide (a moderate effect of about 35% reduction of Sp1-dependent transcription). The next important parameter was the length of the LNA nucleotide. At a concentration of 50 nM, the 19, 16, 13, and 10-base oligonucleotides reduced progesterone receptor gene expression by 91, 86, 46, 33%, respectively. The best result was obtained in the case of the androgen receptor gene attenuation: the concentration required for half-maximal inhibition of transcription (IC50) was estimated by the authors to be 8 nM. Later on, Hu and Corey showed that modification of agPNA-peptide conjugates with hydrophobic amino acids improved their action [133].

Nusinersen [134] is based on the idea to turn off the so-called "intronic splicing switch or splicing silencer" ("ISS") found in the SMN2 gene, regulating the splicing of exons 7 and 8. The resulting protein is capable of replacing the SMN1 protein in terms of its physiological effect. For this purpose, it was proposed to use antisense modified oligonucleotides. Nusinersen is a 2'-O-2-methoxyethyl phosphorothioate ASO. Also, phosphorodiamidate morpholinos (PMO) also attracted significant attention despite the failure of drisapersen. Finally, it was found that 2'-modified RNA PS ASOs with less than four PS in the 3'-terminus showed very good exon 23 skipping efficacy [135].

To date, FDA-approved oligonucleotide drugs have a limited use and are shown below (Table 2) and registered clinical trials in Additional file 1: Table.

### Interactions of artificial DNAs with chromosomes to control gene expression

Many genetic diseases are caused by single gene mutations; and for the purpose of correcting corresponding point mutations, numerous oligo AGPs may be employed, such as pseudocomplementary PNAs (pc-PNA), bifunctional PNA-DNA conjugates (bis-PNA), PNA tail clamp (tc-PNA), and ssPNA. Frequently, in these oligonucleotides, adenine and thymine in PNAs are replaced with diaminopurines and thiouracils, respectively, giving pseudocomplementary PNAs (pcPNAs) that feature increased solubility and affinity [136].

The term "anti-genic PNAs" (agPNAs) is somewhat confusing since it is a nucleic acid mimetic designed to block the function of a particular gene contrary to the immunological meaning of "antigen" that refers to some substance that causes an immune response. The possibility of the formation of a triple helix makes it possible to interfere in the genome at the level of transcriptional regulation, and it is possible to achieve a stronger binding of AGPs to chromosomal DNA, thus disrupting interactions of the latter with the transcriptional, replication or repair machineries.

Although well-designed oligonucleotides made of unmodified DNA can turn off transcription in a very decent degree, PNAs and LNAs are usually superior to unmodified DNAs. Wang and colleagues investigated the possibility of transcription activation by AGPs [137]. The working hypothesis proposed by the authors was that during triple helix formation one of the strands of chromosomal DNA is displaced (the so-called D-loop) and serves as a good target for transcription factors. The work investigated the correlation between the efficiency of transcriptional activation and the size of the D-loop probe. The maximum level of transcription was obtained with the oligos with lengths of 18 monomers.

In 2017, Zaghloul et al. [138] reported on a LNA/RNA gapmer capable of blocking repetitive trinucleotide sequences of chromosomal DNA whose expansion causes Huntington disease. A stronger effect was obtained when phosphorothioate oligonucleotides were used. When fluorescently labeled oligonucleotides were injected into cells, a clear localization of phosphorothioate oligos in the nucleus was shown, whereas phosphodiester oligos were mainly located in the cytoplasm. Apparently, this explains the more pronounced effect of transcription blocking specifically by AGPs.

### Genome editing

In 2005, Andrieu-Soler et al. [139] presented a pioneering work in which a mutation in plasmid transfected into a cell line was corrected using an oligonucleotide containing LNAs at both ends [139]. Later, it was shown that the triple helix formed upon the binding of PNA to DNA stimulates recombination in the cell (co-transfection of TFOs and short, single-stranded DNA donor molecules), [140]. Chin and his co-authors proposed the use of TFO PNAs to induce a frameshift mutation in the same β-globin gene (responsible for β-talassemia) to restore proper splicing of the gene [141]. They improved this approach further in 2012 to correct the hemoglobin gene mutations in hematopoietic cell precursors reaching the probability of correction to 1.63% with PNA and donor DNA versus 0.29% spontaneous correction with donor DNA alone [142].

It should be noted that site-specific mutagenesis and genomic editing with some combination of AGPs seem to have reached the level of a working tool. However, in recent years such an effective, and most importantly, inexpensive, approach as CRISPR-Cas9-mediated genome editing has gained an overwhelming popularity over the AGPs application. This is why there is so little contemporary work on the PNA. Interestingly, Cromwell et al. [143] described the use of AGPs as guide RNAs for the CRISPR-Cas9 systems. LNAs were used as guide RNAs and were found to induce a somewhat slower Cas9 excision-repair activity but accompanied by an increase in selectivity, in other words, reduced off-target editing.

### Delivery to the target inside the living cell and pharmocodynamics in the whole organism

In one of the early works by Hanvey et al. [144], PNAs microinjected into the nucleus successfully suppressed the early region of the SV40 virus. Since then, numerous research groups attempted to optimize intracellular delivery of AGPs into the cell and specific cellular compartments. For this purpose, the conjugation of PNAs with membrane-active peptides is certainly an option. For example, Good et al. [114] showed that a lysine-rich peptide increases the delivery of PNA into bacterial cells up to two orders of magnitude [114]. Oligoarginine may also greatly improve cellular uptake into cancer cells. But the highest delivery rate into the cell was provided by the so-called cell-penetrating peptides (CPPs), the mechanism of action of which has not yet been fully established. As early as 1998, Pooga et al. [145] used CPPs GWTLNSAGYLLGKINLKALAALAKKIL or pAntennapedia (a.k.a. 43–58) conjugated with PNAs for the purpose of reduction of galanin receptor expression in the rat brain.

Dr. Enrica Fabbri and colleagues [146] worked with anti-mir-210 PNA conjugated to oligoarginine (Rpep-PNA-a210 H-RRRRRRR-CCGCTGTCACGCACAG-NH2) using K562 cell culture treated with 15 nM mitramycin to induce the erythroid differentiation. As a result, a decrease in miR-210 levels (measured semi-quantitatively by real-time PCR) was observed concomitantly with the recovery of RAPTOR mRNA levels, which is the target of mir-210.

Brognara et al. [147] described the effect of an oligoarginine-conjugated PNA against miR-221, which is involved in carcinogenesis. Oligoarginine peptides were shown to be effective in improving PNA uptake by cells. The level of miR-221 microRNA in the cells before and after the treatment was measured as well as the recovery of the p27 protein levels, which is the target of miR-221. Notably, elevated levels of microRNA-221 have been observed in many types of cancer including glioma, hepatocellular carcinoma, pancreatic adenocarcinoma, melanoma, chronic lympholeukemia, and papillary thyroid cancer. It is hypothesized that the 27Kip1 24-mer bis-PNA (Anti-miR) can be used to efficiently displace one chain of the miR at the double-stranded RNA stage [148].

Arginincalix[4]arene comprises four arginines linked to a cyclic framework (calixarene[4]), resulting in a bucket-like structure with symmetrical guanidine groups on the rim. These structures have been successfully used as an attractive vehicle for delivering PNA into cells [149]. Arginincalix[4]arene has important advantages: low cellular toxicity to cells, no requirements for covalent bonding with PNA (simple mixing suffices), and protection of PNA from lysosomal degradation upon cellular delivery.

Another interesting approach is a transfection "shuttle” [150]: a deoxythymidine phosphorothioate 8-mer (amphipathic trans-acting polythymidylic thiophosphate triester element—dTtaPS) enters the cell through the energy-dependent mechanism of micropinocytosis and efficiently guides PNA or PMO that are hybridized via a 6 nucleotide-poly-A sequence. This method increases the intracellular delivery of AGPs by a factor of ten. Any PNA can be conjugated to a small poly-A tail to dock to this transfection reagent.

Once inside the cell, AGPs should be transported to a desirable compartment. In 2000, Chinnery et al. [151] demonstrated a successful intracellular targeting of PNA to mitochondria. This PNA contained a peptide sequence from the localization signal of cytochrome c oxidase subunit VIII. A nuclear localization signal ("NLS") fused to the PNA can be used for the purpose of nucleus-specific targeting [152]. The successful synthesis scheme of a modified PNA that mimics the nuclear localization signal was reported by Sforza et al. [153].

It was shown that the conjugation of PNA with short basic peptides such as octa(L-lysine) allowed the resulting conjugate to accumulate in the liver, kidneys, and adipose tissue rich in fat [154]. Based on this notion, the authors suggested that it may be possible to deliver PNAs into the cells with abnormally high content of fat: for example, the amount of lipid accumulation in the form of lipid droplets is significantly increased in hepatocytes of obese liver patients and in striated muscles in animal models of type 2 diabetes.

Most difficulties with the delivery of AGPs arise from the fact that oligonucleotides are highly hydrophilic polyanions (M_r_ of a typical ASOs in the range of 4–10 kDa, and ds-siRNAs around 14 kDa, they cannot pass cellular membranes without assistance). Although PNA is much less hydrophilic than “true” nucleic acids, this problem still pertains, especially for therapeutical needs: DNA/RNA or AGPs upon entering bloodstream should withstand nucleases, and neutralization by sequestration by major plasma proteins such as IgM, evade clearance in the kidneys, as well as the opsonization effect in the liver by Kupffer cells and other phagocytes. Finally, they should be able to penetrate blood vessel walls in targeted organs. Moreover, neither naked DNA/RNA nor PNA can penetrate through the blood–brain barrier. For these reasons, major successes with therapeutic oligonucleotides are limited to those used in local delivery (for example, to the eye), and to the liver. Excellent reviews may be useful for in-depth reading [154, 155].

Another very important issue is the rate of decay of natural and modified nucleic acids inside the cell and in the bodily fluids, in other words, their pharmacokinetic stability. Shortly after the first development of PNA, Demidov et al. [156] reported that in the cytoplasmic extract of eukaryotic cells no more than 20% of PNA decayed during 2 h of incubation. It was also indicated that PNA H-T5-LysNH2 degrades 30 times slower than the control peptide (FWYCFWYKFWYK-OH) under the action of peptidase and 1000 times slower when treated with Proteinase K. Despite this early report, PNAs are deemed stable inside the cell. Distribution in tissues and pharmacokinetics of PNA in the whole organism have been studied in more detail by McMahon et al. [157]. It was shown that after injection into the tail vein, the elimination half-life was about 17 min for PNA in the rat, and about 90% of PNA is excreted unchanged into urine within 24 h [158].

LNA and their analogues (amino-LNA, thio-LNA and α-L-LNA) were studied in terms of stability and tissue distribution by Fluiter et al. [158]. It was found that these LNAs are very stable in the serum and there is some affinity of amino-LNA to the heart, liver, and lungs, as compared to other LNA types [159]. Borgatti et al. [157] investigated the biophysical properties of LNA/DNA chimeras as SP1 and NF-κb transcription factor decoys with the focus on the resistance of these molecules to various enzymes and found that these PNA/DNA chimeras were completely resistant to 3'-5' exonucleases, and also much more resistant to 5'-3' exonucleases and DNase I than the corresponding unmodified oligonucleotides.

The half-life of the nucleic acid mimetic drug Nusinersen/Spinraza® (a 2'-O-2-methoxyethyl phosphorothioate oligonucleotide; used by intrathecal injection directly into the CSF) is from 135 to 177 days in CSF and from 63 to 87 days in plasma, where it slowly leaks from CSF. Nusinersen/Spinraza® is metabolized by 5’- and 3’-exonucleases, however, the primary route of elimination is urinary excretion.

Both pharmacodynamic and pharmacokinetic parameters may be significantly improved by conjugation with other carrier molecules and nanoparticles, liposomes, carbon based nanocarriers, as well as supramolecular self-assembly (recently reviewed in detail by Volpi et al. [159]). On a related note, Ma et al. [160] have reported a successful application of nanoparticles made of nano-sized porous silicon for disulfide-conjugated PNAs in such a way that after the particles are absorbed into the cytoplasm, the release of PNA was achieved by the action of cellular glutathione.

### Immune response

Undesirable anti-drug immune response poses serious problems, for example, anti-drug antibodies (ADAs) were detected in 6% of patients treated with Nusinersen*/*Spinraza*®*. Both high molecular weight DNA and synthetic oligonucleotides rich in unmethylated CpG dinucleotides induce proliferation and the secretion of immunoglobulins by B cells [161] through toll-like receptor (TLR) signaling. Thus, CpG methylation in higher eukaryotes is a vital part of the friend-or-foe recognition by the immune system, since bacterial DNA usually possesses low levels of CpG methylation. Hence, the lack of proper methylation in therapeutic oligonucleotides poses a serious problem. Vollmer et al. [162] evaluated the ability of phosphorothioate chimeric LNA/DNA-antisense oligonucleotides to induce the innate immune response in B-cell lines using sequences recognized by human TLR9. LNA was shown to be much less stimulatory especially when native nucleotides were replaced with G-LNA or C-LNA in CpG dinucleotides. Oligonucleotides with CpG islands composed of LNA demonstrated only slight activation of IL-6, while IL-10, IFN-α and IL-10 remained virtually non-induced. Later, Lange et al. [163] investigated the similar ability TNAs to activate B-cells. It was shown in a human B-cell line that TNA oligonucleotides carrying unmethylated CpG islets are able to induce a full immune response, although to a lesser extent than the corresponding natural DNA. This is consistent with what we discussed earlier: AGPs are not always very good substitutes for nucleic acids as interactors with proteins but, fortunately, sometimes this provides exciting benefits for their practical applications.

### AGPs in oncology

As there are numerous reports on the successful applications of AGPs in various oncological models, we should mention only one here to give a representative example. LNAs were introduced at the 3'-ends of oligonucleotides mimicking G-quadruplex decoys in order to knockdown KRAS in pancreatic cancer cells. Some of these strongly suppressed KRAS expression in Panc-1, pancreatic cancer cells, and reduced the tumor xenograft growth in immunodeficient mice, increasing also their median survival time by 70% [164]. Yet, early hopes [164, 165] for anti-cancer AGPs were clearly exaggerated: problems such as poor delivery, low stability, or toxicity, were severe impediments, only partly resolved for two organs: liver and eye. The diagnostic use of LNAs in oncology was reviewed in [166].

Notably, Falanga and colleagues [167] described a comprehensive approach to create a novel anti-cancer agent based on a PNA which is capable of blocking the transcription of the pro-apoptotic gene Bcl-2. It binds to the G-rich sequence, located 52–30 bp upstream of the P1 promoter of the gene and blocks the formation of the transcriptional complex.

An example of creating an anticancer drug that disrupts mitochondrial genome expression can be found in the work of Shen-Sun Chen and colleagues [168]. The 5'-CAGACCGCCCCAAAAGA-3' PNA with triphenylphosphonium attached to facilitate its targeted delivery was shown to bind the mitochondrial DNA. The binding was in the D-loop region where the promoters of mitochondrial heavy and light DNA chains are located. Authors have shown a significant (almost threefold) decrease in the growth rate of xenograft tumors following daily intratumoral injections of the preparation, in comparison to the control preparation containing nonspecific PNA of the same size.

An expanding arsenal of available chemical modifications holds promise for significant improvements of AGPs in oncology, as, for example, it was demonstrated with mesyl phosphoramidates (more potent that PSOs [21]).

### Other modifications: nucleobases and termini

The number of reports on modifications of nucleobases is overwhelming. Thus, it impossible to review all of them in this format. However, we should remember that C-5 of pyrimidines is the most popular position for various modifications. This is due to a significant tolerability of DNA polymerases to such substitutions. We should only briefly mention the most interesting attempts to endow AGPs with certain unusual properties. This includes those modifications that allow irreversible complex formation through covalent cross-linking under physiological conditions: attachment of furan moieties [169], and nucleobases that allow bridging upon complexation with metal ions [170].

For some chemical substances stability can be increased through the use of kinetic isotope effect by substitution of deuteriums for protiums at a critical position where C-H bond cleavage is the rate-limiting step in the reaction mechanism. Unfortunately, this is not possible in DNA or RNA backbones to protect them from nucleases, however, in several cases the nitrogenous bases can be reinforced to a certain degree by using this approach, for example, methylation of cytosine by DNA methyltransferase 3A can be decelerated to some extent by deuterium substitutions for C5 and C6 hydrogens [171].

Terminal modifications of native nucleic acids or AGPs are innumerous. We, however, would like to mention one of special importance: branching in various degrees including dendromerization. From early synthetic attempts [172] this field achieved recognition for many specialized applications (reviewed in [173]).

## Conclusion and future perspectives

AGPs became a powerful tool for the regulation of gene expression and potentially for genome editing. Their usefulness for the regulation of intricate cellular processes is becoming more and more obvious with each passing decade. Occupying an intermediate position between small molecules and large molecular machines such as CRISPR-Cas9 complexes, AGPs may ultimately evolve into ultra-precise personalized weapons against diseases as diverse as parasitic infections, inherited disorders, and cancer. These applications may expand the limits of the current use of AGPs in diagnostics that are already state of the art to applications in theranostics (includes both diagnostic and therapeutic approaches).

However, to reach these goals, a lot of optimization steps are required. Applications of AGPs will be further improved through the use of special dedicated software [174], detection of PNAs using nanopore platforms [175], and with rational improvements in reducing side effects such as hepatotoxicity [176]. Machine learning algorithms partially help alleviate this problem by predicting possible side effects of experimental drugs, however, a lot of work needs to be done in this direction [177–179]

In terms of exciting perspectives that may open sometimes for oncological applications of AGPs, arming of oncolytic viruses with transgenes encoding AGP-synthesizing polymerases inside the cancer cells would be of great interest, since such an approach may greatly boost the action of cytotoxic drugs.

## Supplementary Information

**Additional file 1**. Clinical trials of oligonucleotide based therapeutics.

## Data Availability

Not applicable.

## References

[1] Anosova I, Kowal EA, Dunn MR, Chaput JC, Van Horn WD, Egli M (2016). The structural diversity of artificial genetic polymers. Nucleic Acids Res.

[2] Moccia M, Adamo MFA, Saviano M (2014). Insights on chiral, backbone modified peptide nucleic acids: properties and biological activity. Artif DNA PNA XNA.

[3] Barluenga S, Winssinger N (2015). PNA as a biosupramolecular tag for programmable assemblies and reactions. Acc Chem Res.

[4] Pinheiro VB, Holliger P (2014). Towards XNA nanotechnology: new materials from synthetic genetic polymers. Trends Biotechnol.

[5] Chen T, Hongdilokkul N, Liu Z, Thirunavukarasu D, Romesberg FE (2016). The expanding world of DNA and RNA. Curr Opin Chem Biol.

[6] Das A, Pradhan B (2021). Evolution of peptide nucleic acid with modifications of its backbone and application in biotechnology. Chem Biol Drug Des.

[7] Liczner C, Duke K, Juneau G, Egli M, Wilds CJ (2021). Beyond ribose and phosphate: Selected nucleic acid modifications for structure-function investigations and therapeutic applications. Beilstein J Org Chem.

[8] Tu T, Huan S, Ke G, Zhang X (2022). Functional xeno nucleic acids for biomedical application. Chem Res Chin Univ.

[9] Wang F, Li P, Chu HC, Lo PK (2022). Nucleic acids and their analogues for biomedical applications. Biosensors (Basel).

[10] Taylor AI, Houlihan G, Holliger P (2019). Beyond DNA and RNA: the expanding toolbox of synthetic genetics. Cold Spring Harb Perspect Biol.

[11] Chaput JC, Herdewijn P, Hollenstein M (2020). Orthogonal genetic systems. ChemBioChem.

[12] Chaput JC (2021). Redesigning the genetic polymers of life. Acc Chem Res.

[13] Asanuma H, Kamiya Y, Kashida H, Murayama K (2022). Xeno nucleic acids (XNAs) having non-ribose scaffolds with unique supramolecular properties. Chem Commun (Camb).

[14] Hebel M, Riegger A, Zegota MM, Kizilsavas G, Gačanin J, Pieszka M (2019). Sequence programming with dynamic boronic acid/catechol binary codes. J Am Chem Soc.

[15] Eschenmoser A, Loewenthal E (1992). Chemistry of potentially prebiological natural products. Chem Soc Rev.

[16] Letsinger RL, Bach SA, Eadie JS (1986). Effects of pendant groups at phosphorus on binding properties of d-ApA analogues. Nucleic Acids Res.

[17] Crooke ST, Vickers TA, Liang X (2020). Phosphorothioate modified oligonucleotide–protein interactions. Nucleic Acids Res.

[18] Tomaszewska-Antczak A, Jastrzębska K, Maciaszek A, Mikołajczyk B, Guga P (2018). P-Stereodefined phosphorothioate analogs of glycol nucleic acids-synthesis and structural properties. RSC Adv.

[19] Hagedorn PH, Yakimov V, Ottosen S, Kammler S, Nielsen NF, Høg AM (2013). Hepatotoxic potential of therapeutic oligonucleotides can be predicted from their sequence and modification pattern. Nucleic Acid Ther.

[20] Miroshnichenko SK, Patutina OA, Burakova EA, Chelobanov BP, Fokina AA, Vlassov VV (2019). Mesyl phosphoramidate antisense oligonucleotides as an alternative to phosphorothioates with improved biochemical and biological properties. Proc Natl Acad Sci U S A.

[21] Patutina OA, Gaponova Miroshnichenko SK, Sen’kova AV, Savin IA, Gladkikh DV, Burakova EA (2020). Mesyl phosphoramidate backbone modified antisense oligonucleotides targeting miR-21 with enhanced in vivo therapeutic potency. Proc Natl Acad Sci U S A.

[22] Teplova M, Minasov G, Tereshko V, Inamati GB, Cook PD, Manoharan M (1999). Crystal structure and improved antisense properties of 2’-O-(2-methoxyethyl)-RNA. Nat Struct Biol.

[23] Honcharenko D, Rocha CSJ, Lundin KE, Maity J, Milton S, Tedebark U (2022). 2′-O-(N-(Aminoethyl)carbamoyl)methyl modification allows for lower phosphorothioate content in splice-switching oligonucleotides with retained activity. Nucleic Acid Ther.

[24] Peng CG, Damha MJ (2007). Polymerase-directed synthesis of 2’-deoxy-2’-fluoro-beta-D-arabinonucleic acids. J Am Chem Soc.

[25] Wang Z, Xu W, Liu L, Zhu TF (2016). A synthetic molecular system capable of mirror-image genetic replication and transcription. Nat Chem.

[26] Nielsen PE, Egholm M, Berg RH, Buchardt O (1991). Sequence-selective recognition of DNA by strand displacement with a thymine-substituted polyamide. Science.

[27] Egholm M, Buchardt O, Christensen L, Behrens C, Freier SM, Driver DA (1993). PNA hybridizes to complementary oligonucleotides obeying the Watson-Crick hydrogen-bonding rules. Nature.

[28] Zengeya T, Gindin A, Rozners E (2013). Improvement of sequence selectivity in triple helical recognition of RNA by phenylalanine-derived PNA. Artif DNA PNA XNA.

[29] Kumar V, Brodyagin N, Rozners E (2020). Triplex-forming peptide nucleic acids with extended backbones. ChemBioChem.

[30] Govindaraju T, Kumar VA (2005). Backbone-extended pyrrolidine peptide nucleic acids (bepPNA): design, synthesis and DNA/RNA binding studies. Chem Commun (Camb).

[31] Vilaivan T, Lowe G (2002). A novel pyrrolidinyl PNA showing high sequence specificity and preferential binding to DNA over RNA. J Am Chem Soc.

[32] Vilaivan C, Srisuwannaket C, Ananthanawat C, Suparpprom C, Kawakami J, Yamaguchi Y (2011). Pyrrolidinyl peptide nucleic acid with α/β-peptide backbone: a conformationally constrained PNA with unusual hybridization properties. Artif DNA PNA XNA.

[33] Vilaivan T (2015). Pyrrolidinyl PNA with α/β-dipeptide backbone: from development to applications. Acc Chem Res.

[34] Zheng H, Botos I, Clausse V, Nikolayevskiy H, Rastede EE, Fouz MF (2021). Conformational constraints of cyclopentane peptide nucleic acids facilitate tunable binding to DNA. Nucleic Acids Res.

[35] Govindaraju T, Madhuri V, Kumar VA, Ganesh KN (2006). Cyclohexanyl peptide nucleic acids (chPNAs) for preferential RNA binding: effective tuning of dihedral angle beta in PNAs for DNA/RNA discrimination. J Org Chem.

[36] Summerton JE, Weller DD. Uncharged morpholino-based polymers having achiral intersubunit linkages. 1991 [cited 2022 Jun 22]. Available from: https://patents.google.com/patent/US5034506A/en

[37] Pitsch S, Krishnamurthy R, Bolli M, Wendeborn S, Holzner A, Minton M (1995). Pyranosyl-RNA (‘p-RNA’): base-pairing selectivity and potential to replicate. Helv Chim Acta.

[38] Beier M, Reck F, Wagner T, Krishnamurthy R, Eschenmoser A (1999). Chemical etiology of nucleic acid structure: comparing pentopyranosyl-(2’–>4’) oligonucleotides with RNA. Science.

[39] Reck F, Wippo H, Kudick R, Bolli M, Ceulemans G, Krishnamurthy R (1999). l-α-Lyxopyranosyl (4‘→3‘) oligonucleotides: a base-pairing system containing a shortened backbone1. Org Lett.

[40] Schöning K, Scholz P, Guntha S, Wu X, Krishnamurthy R, Eschenmoser A (2000). Chemical etiology of nucleic acid structure: the alpha-threofuranosyl-(3’–>2’) oligonucleotide system. Science.

[41] Liu C, Cozens C, Jaziri F, Rozenski J, Maréchal A, Dumbre S (2018). Phosphonomethyl oligonucleotides as backbone-modified artificial genetic polymers. J Am Chem Soc.

[42] Hajjar M, Chim N, Liu C, Herdewijn P, Chaput JC (2022). Crystallographic analysis of engineered polymerases synthesizing phosphonomethylthreosyl nucleic acid. Nucleic Acids Res.

[43] Ng P-S, Bergstrom DE (2005). Alternative nucleic acid analogues for programmable assembly: hybridization of LNA to PNA. Nano Lett.

[44] D’Alonzo D, Amato J, Schepers G, Froeyen M, Van Aerschot A, Herdewijn P (2013). Enantiomeric selection properties of β-homoDNA: enhanced pairing for heterochiral complexes. Angew Chem Int Ed Engl.

[45] Campbell MA, Wengel J (2011). Locked vs. unlocked nucleic acids (LNA vs. UNA): contrasting structures work towards common therapeutic goals. Chem Soc Rev.

[46] Zhang L, Peritz A, Meggers E (2005). A simple glycol nucleic acid. J Am Chem Soc.

[47] Lescrinier E, Esnouf R, Schraml J, Busson R, Heus H, Hilbers C (2000). Solution structure of a HNA-RNA hybrid. Chem Biol.

[48] Baraniak D, Boryski J (2021). Triazole-modified nucleic acids for the application in bioorganic and medicinal chemistry. Biomedicines.

[49] Petersen M, Bondensgaard K, Wengel J, Jacobsen JP (2002). Locked nucleic acid (LNA) recognition of RNA: NMR solution structures of LNA:RNA hybrids. J Am Chem Soc.

[50] Moreno PMD, Geny S, Pabon YV, Bergquist H, Zaghloul EM, Rocha CSJ (2013). Development of bis-locked nucleic acid (bisLNA) oligonucleotides for efficient invasion of supercoiled duplex DNA. Nucleic Acids Res.

[51] Geny S, Moreno PMD, Krzywkowski T, Gissberg O, Andersen NK, Isse AJ (2016). Next-generation bis-locked nucleic acids with stacking linker and 2’-glycylamino-LNA show enhanced DNA invasion into supercoiled duplexes. Nucleic Acids Res.

[52] Felsenfeld G, Davies DR, Rich A (1957). Formation of a three-stranded polynucleotide molecule. J Am Chem Soc.

[53] Felsenfeld G, Rich A (1957). Studies on the formation of two- and three-stranded polyribonucleotides. Biochem Biophys Acta.

[54] Hoogsteen K (1963). The crystal and molecular structure of a hydrogen-bonded complex between 1-methylthymine and 9-methyladenine. Acta Crystallogr A.

[55] Lesnik E (1997). Triplex formation between DNA and mixed purine-pyrimidine PNA analog with lysines in backbone. Nucleosides Nucleotides.

[56] Bukanov NO, Demidov VV, Nielsen PE, Frank-Kamenetskii MD (1998). PD-loop: a complex of duplex DNA with an oligonucleotide. Proc Natl Acad Sci U S A.

[57] Bentin T, Larsen HJ, Nielsen PE (2003). Combined triplex/duplex invasion of double-stranded DNA by “tail-clamp” peptide nucleic acid. Biochemistry.

[58] Solé A, Delagoutte E, Ciudad CJ, Noé V, Alberti P (2017). Polypurine reverse-Hoogsteen (PPRH) oligonucleotides can form triplexes with their target sequences even under conditions where they fold into G-quadruplexes. Sci Rep.

[59] Xu Y, Gissberg O, Pabon-Martinez YV, Wengel J, Lundin KE, Smith CIE (2019). The ability of locked nucleic acid oligonucleotides to pre-structure the double helix: a molecular simulation and binding study. PLoS ONE.

[60] Panyutin IG, Onyshchenko MI, Englund EA, Appella DH, Neumann RD (2012). Targeting DNA G-quadruplex structures with peptide nucleic acids. Curr Pharm Des.

[61] Eriksson M, Nielsen PE (1996). Solution structure of a peptide nucleic acid-DNA duplex. Nat Struct Biol.

[62] Brown SC, Thomson SA, Veal JM, Davis DG (1994). NMR solution structure of a peptide nucleic acid complexed with RNA. Science.

[63] Ban C, Ramakrishnan B, Sundaralingam M (1994). A single 2’-hydroxyl group converts B-DNA to A-DNA. Crystal structure of the DNA-RNA chimeric decamer duplex d(CCGGC)r(G)d(CCGG) with a novel intermolecular G-C base-paired quadruplet. J Mol Biol.

[64] Demidov V, Frank-Kamenetskii MD, Egholm M, Buchardt O, Nielsen PE (1993). Sequence selective double strand DNA cleavage by peptide nucleic acid (PNA) targeting using nuclease S1. Nucleic Acids Res.

[65] Bergmann F, Bannwarth W, Tam S (1995). Solid phase synthesis of directly linked PNA-DNA-hybrids. Tetrahedron Lett.

[66] Lyamichev VI, Mirkin SM, Frank-Kamenetskii MD (1986). Structures of homopurine-homopyrimidine tract in superhelical DNA. J Biomol Struct Dyn.

[67] Cheng AJ, Van Dyke MW (1993). Monovalent cation effects on intermolecular purine-purine-pyrimidine triple-helix formation. Nucleic Acids Res.

[68] Egholm M, Christensen L, Dueholm KL, Buchardt O, Coull J, Nielsen PE (1995). Efficient pH-independent sequence-specific DNA binding by pseudoisocytosine-containing bis-PNA. Nucleic Acids Res.

[69] Kim K-H, Nielsen PE, Glazer PM (2007). Site-directed gene mutation at mixed sequence targets by psoralen-conjugated pseudo-complementary peptide nucleic acids. Nucleic Acids Res.

[70] Demidov VV, Bukanov NO, Frank-Kamenetskii D (2000). Duplex DNA capture. Curr Issues Mol Biol.

[71] Smolina IV, Kuhn H, Lee C, Frank-Kamenetskii MD (2008). Fluorescence-based detection of short DNA sequences under non-denaturing conditions. Bioorg Med Chem.

[72] Ahn JJ, Kim Y, Lee SY, Hong JY, Kim GW, Hwang SY (2015). Fluorescence melting curve analysis using self-quenching dual-labeled peptide nucleic acid probes for simultaneously identifying multiple DNA sequences. Anal Biochem.

[73] Jeon SH, Kim HW, Kim BN, Kang N, Yeo CD, Park CK (2019). Comparison of PNA clamping-assisted fluorescence melting curve analysis and PNA clamping in detecting EGFR mutations in matched tumor tissue, cell block, pleural effusion and blood of lung cancer patients with malignant pleural effusion. In Vivo.

[74] Igloi GL (1998). Variability in the stability of DNA-peptide nucleic acid (PNA) single-base mismatched duplexes: real-time hybridization during affinity electrophoresis in PNA-containing gels. Proc Natl Acad Sci U S A.

[75] Choi J-J, Jang M, Kim J, Park H (2010). Highly sensitive PNA array platform technology for single nucleotide mismatch discrimination. J Microbiol Biotechnol.

[76] You Y, Moreira BG, Behlke MA, Owczarzy R (2006). Design of LNA probes that improve mismatch discrimination. Nucleic Acids Res.

[77] Le BT, Adams AM, Fletcher S, Wilton SD, Veedu RN (2017). Rational design of short locked nucleic acid-modified 2’-O-methyl antisense oligonucleotides for efficient exon-skipping in vitro. Mol Ther Nucleic Acids.

[78] Agerholm IE, Ziebe S, Williams B, Berg C, Crüger DG, Petersen GB (2005). Sequential FISH analysis using competitive displacement of labelled peptide nucleic acid probes for eight chromosomes in human blastomeres. Hum Reprod.

[79] Giunta S, Funabiki H (2017). Integrity of the human centromere DNA repeats is protected by CENP-A, CENP-C, and CENP-T. Proc Natl Acad Sci U S A.

[80] Beliveau BJ, Boettiger AN, Avendaño MS, Jungmann R, McCole RB, Joyce EF (2015). Single-molecule super-resolution imaging of chromosomes and in situ haplotype visualization using Oligopaint FISH probes. Nat Commun.

[81] Stender H, Mollerup TA, Lund K, Petersen KH, Hongmanee P, Godtfredsen SE (1999). Direct detection and identification of Mycobacterium tuberculosis in smear-positive sputum samples by fluorescence in situ hybridization (FISH) using peptide nucleic acid (PNA) probes. Int J Tuberc Lung Dis.

[82] Lefmann M, Schweickert B, Buchholz P, Göbel UB, Ulrichs T, Seiler P (2006). Evaluation of peptide nucleic acid-fluorescence in situ hybridization for identification of clinically relevant mycobacteria in clinical specimens and tissue sections. J Clin Microbiol.

[83] Oliveira K, Haase G, Kurtzman C, Hyldig-Nielsen JJ, Stender H (2001). Differentiation of Candida albicans and Candida dubliniensis by fluorescent in situ hybridization with peptide nucleic acid probes. J Clin Microbiol.

[84] Ishige T, Itoga S, Matsushita K, Nomura F (2016). Locked nucleic acid probe enhances Sanger sequencing sensitivity and improves diagnostic accuracy of high-resolution melting-based KRAS mutational analysis. Clin Chim Acta.

[85] Astakhova IK, Wengel J (2014). Scaffolding along nucleic acid duplexes using 2’-amino-locked nucleic acids. Acc Chem Res.

[86] Bat-Ochir C, Kim Y-S, Kim HG, Lee SS, Lee HW, Park HK (2021). Development, evaluation of the PNA RT-LAMP assay for rapid molecular detection of SARS-CoV-2. Sci Rep.

[87] Singh KR, Sridevi P, Singh RP (2020). Potential applications of peptide nucleic acid in biomedical domain. Eng Rep.

[88] Nulf CJ, Corey DR (2002). DNA assembly using bis-peptide nucleic acids (bisPNAs). Nucleic Acids Res.

[89] Berger O, Gazit E (2017). Molecular self-assembly using peptide nucleic acids. Biopolymers.

[90] Kumar S, Pearse A, Liu Y, Taylor RE (2020). Modular self-assembly of gamma-modified peptide nucleic acids in organic solvent mixtures. Nat Commun.

[91] Becker AL, Johnston APR, Caruso F (2010). Peptide nucleic acid films and capsules: assembly and enzymatic degradation. Macromol Biosci.

[92] Iverson D, Serrano C, Brahan AM, Shams A, Totsingan F, Bell AJ (2015). Characterization of the structural and protein recognition properties of hybrid PNA-DNA four-way junctions. Arch Biochem Biophys.

[93] Brown ZZ, Müller MM, Kong HE, Lewis PW, Muir TW (2015). Targeted histone peptides: insights into the spatial regulation of the methyltransferase PRC2 by using a surrogate of heterotypic chromatin. Angew Chem Int Ed Engl.

[94] Boffa LC, Carpaneto EM, Allfrey VG (1995). Isolation of active genes containing CAG repeats by DNA strand invasion by a peptide nucleic acid. Proc Natl Acad Sci U S A.

[95] Edayathumangalam RS, Weyermann P, Gottesfeld JM, Dervan PB, Luger K (2004). Molecular recognition of the nucleosomal “supergroove”. Proc Natl Acad Sci U S A.

[96] Gottesfeld JM, Melander C, Suto RK, Raviol H, Luger K, Dervan PB (2001). Sequence-specific recognition of DNA in the nucleosome by pyrrole-imidazole polyamides. J Mol Biol.

[97] Ryoo S-R, Lee J, Yeo J, Na H-K, Kim Y-K, Jang H (2013). Quantitative and multiplexed microRNA sensing in living cells based on peptide nucleic acid and nano graphene oxide (PANGO). ACS Nano.

[98] Parfenyev S, Singh A, Fedorova O, Daks A, Kulshreshtha R, Barlev NA (2021). Interplay between p53 and non-coding RNAs in the regulation of EMT in breast cancer. Cell Death Dis.

[99] Candi E, Amelio I, Agostini M, Melino G (2015). MicroRNAs and p63 in epithelial stemness. Cell Death Differ.

[100] Feng Z, Ye Z, Xie J, Chen W, Li W, Xing C (2021). Study on the mechanism of LOXL1-AS1/miR-3614-5p/YY1 signal axis in the malignant phenotype regulation of hepatocellular carcinoma. Biol Direct.

[101] Qian J, Lei X, Sun Y, Zheng L, Li J, Zhang S (2021). Long non-coding RNA SNHG8 enhances triple-negative breast cancer cell proliferation and migration by regulating the miR-335-5p/PYGO2 axis. Biol Direct.

[102] Elmén J, Lindow M, Schütz S, Lawrence M, Petri A, Obad S (2008). LNA-mediated microRNA silencing in non-human primates. Nature.

[103] Musumeci D, Bucci EM, Roviello GN, Sapio R, Valente M, Moccia M (2011). DNA-based strategies for blocking HMGB1 cytokine activity: design, synthesis and preliminary in vitro/in vivo assays of DNA and DNA-like duplexes. Mol Biosyst.

[104] Mischiati C, Borgatti M, Bianchi N, Rutigliano C, Tomassetti M, Feriotto G (1999). Interaction of the human NF-kappaB p52 transcription factor with DNA-PNA hybrids mimicking the NF-kappaB binding sites of the human immunodeficiency virus type 1 promoter. J Biol Chem.

[105] Borgatti M, Breda L, Cortesi R, Nastruzzi C, Romanelli A, Saviano M (2002). Cationic liposomes as delivery systems for double-stranded PNA-DNA chimeras exhibiting decoy activity against NF-kappaB transcription factors. Biochem Pharmacol.

[106] Finotti A, Borgatti M, Bezzerri V, Nicolis E, Lampronti I, Dechecchi M (2012). Effects of decoy molecules targeting NF-kappaB transcription factors in Cystic fibrosis IB3-1 cells: recruitment of NF-kappaB to the IL-8 gene promoter and transcription of the IL-8 gene. Artif DNA PNA XNA.

[107] Borgatti M, Romanelli A, Saviano M, Pedone C, Lampronti I, Breda L (2003). Resistance of decoy PNA-DNA chimeras to enzymatic degradation in cellular extracts and serum. Oncol Res.

[108] Borgatti M, Lampronti I, Romanelli A, Pedone C, Saviano M, Bianchi N (2003). Transcription factor decoy molecules based on a peptide nucleic acid (PNA)-DNA chimera mimicking Sp1 binding sites. J Biol Chem.

[109] Wahlestedt C, Salmi P, Good L, Kela J, Johnsson T, Hökfelt T (2000). Potent and nontoxic antisense oligonucleotides containing locked nucleic acids. Proc Natl Acad Sci U S A.

[110] Shammas MA, Simmons CG, Corey DR, Shmookler Reis RJ (1999). Telomerase inhibition by peptide nucleic acids reverses “immortality” of transformed human cells. Oncogene.

[111] Greenberger LM, Horak ID, Filpula D, Sapra P, Westergaard M, Frydenlund HF (2008). A RNA antagonist of hypoxia-inducible factor-1alpha, EZN-2968, inhibits tumor cell growth. Mol Cancer Ther.

[112] Swayze EE, Siwkowski AM, Wancewicz EV, Migawa MT, Wyrzykiewicz TK, Hung G (2007). Antisense oligonucleotides containing locked nucleic acid improve potency but cause significant hepatotoxicity in animals. Nucleic Acids Res.

[113] Burdick AD, Sciabola S, Mantena SR, Hollingshead BD, Stanton R, Warneke JA (2014). Sequence motifs associated with hepatotoxicity of locked nucleic acid–modified antisense oligonucleotides. Nucleic Acids Res.

[114] Good L, Awasthi SK, Dryselius R, Larsson O, Nielsen PE (2001). Bactericidal antisense effects of peptide-PNA conjugates. Nat Biotechnol.

[115] Good L, Nielsen PE (1998). Inhibition of translation and bacterial growth by peptide nucleic acid targeted to ribosomal RNA. Proc Natl Acad Sci U S A.

[116] Kiran D, Sriranganathan N (2014). The antimicrobial effect of anti-dnaK peptide nucleic acids on multidrug resistant strains of Escherichia coli and Salmonella enterica serovar Typhimurium. Beta Beta Beta Biol Soc.

[117] Kolevzon N, Nasereddin A, Naik S, Yavin E, Dzikowski R (2014). Use of peptide nucleic acids to manipulate gene expression in the malaria parasite Plasmodium falciparum. PLoS ONE.

[118] Amit-Avraham I, Pozner G, Eshar S, Fastman Y, Kolevzon N, Yavin E (2015). Antisense long noncoding RNAs regulate var gene activation in the malaria parasite Plasmodium falciparum. Proc Natl Acad Sci U S A.

[119] Iubatti M, Gabas IM, Cavaco LM, Mood EH, Lim E, Bonanno F (2022). Antisense peptide nucleic acid-diaminobutanoic acid dendron conjugates with SbmA-Independent antimicrobial activity against gram-negative bacteria. ACS Infect Dis.

[120] Kesy J, Patil KM, Kumar SR, Shu Z, Yong HY, Zimmermann L (2019). A short chemically modified dsRNA-binding PNA (dbPNA) inhibits influenza viral replication by targeting viral RNA panhandle structure. Bioconjug Chem.

[121] Dominski Z, Kole R (1993). Restoration of correct splicing in thalassemic pre-mRNA by antisense oligonucleotides. Proc Natl Acad Sci U S A.

[122] Dunckley MG, Manoharan M, Villiet P, Eperon IC, Dickson G (1998). Modification of splicing in the dystrophin gene in cultured Mdx muscle cells by antisense oligoribonucleotides. Hum Mol Genet.

[123] Karras JG, McKay RA, Dean NM, Monia BP (2000). Deletion of individual exons and induction of soluble murine interleukin-5 receptor-alpha chain expression through antisense oligonucleotide-mediated redirection of pre-mRNA splicing. Mol Pharmacol.

[124] Shiraishi T, Eysturskarth J, Nielsen PE (2010). Modulation of mdm2 pre-mRNA splicing by 9-aminoacridine-PNA (peptide nucleic acid) conjugates targeting intron-exon junctions. BMC Cancer.

[125] Althubiti M, Rada M, Samuel J, Escorsa JM, Najeeb H, Lee K-G (2016). BTK modulates p53 activity to enhance apoptotic and senescent responses. Cancer Res.

[126] Davidovich P, Aksenova V, Petrova V, Tentler D, Orlova D, Smirnov S (2015). Discovery of novel isatin-based p53 inducers. ACS Med Chem Lett.

[127] Fedorova O, Daks A, Petrova V, Petukhov A, Lezina L, Shuvalov O (2018). Novel isatin-derived molecules activate p53 via interference with Mdm2 to promote apoptosis. Cell Cycle.

[128] Sazonova EV, Petrichuk SV, Kopeina GS, Zhivotovsky B (2021). A link between mitotic defects and mitotic catastrophe: detection and cell fate. Biol Direct.

[129] Rozenberg JM, Zvereva S, Dalina A, Blatov I, Zubarev I, Luppov D (2021). The p53 family member p73 in the regulation of cell stress response. Biol Direct.

[130] Panatta E, Zampieri C, Melino G, Amelio I (2021). Understanding p53 tumour suppressor network. Biol Direct.

[131] Wancewicz EV, Maier MA, Siwkowski AM, Albertshofer K, Winger TM, Berdeja A (2010). Peptide nucleic acids conjugated to short basic peptides show improved pharmacokinetics and antisense activity in adipose tissue. J Med Chem.

[132] Beane RL, Ram R, Gabillet S, Arar K, Monia BP, Corey DR (2007). Inhibiting gene expression with locked nucleic acids (LNAs) that target chromosomal DNA. Biochemistry.

[133] Hu J, Corey DR (2007). Inhibiting gene expression with peptide nucleic acid (PNA)–peptide conjugates that target chromosomal DNA. Biochemistry.

[134] Singh RN, Singh NN, Singh NK, Androphy EJ. Spinal muscular atrophy (SMA) treatment via targeting of SMN2 splice site inhibitory sequences. 2010 [cited 2022 Jun 21]. Available from: https://patents.google.com/patent/US7838657B2/en.

[135] Le BT, Agarwal S, Veedu RN (2021). Evaluation of DNA segments in 2’-modified RNA sequences in designing efficient splice switching antisense oligonucleotides. RSC Adv.

[136] Demidov VV, Protozanova E, Izvolsky KI, Price C, Nielsen PE, Frank-Kamenetskii MD (2002). Kinetics and mechanism of the DNA double helix invasion by pseudocomplementary peptide nucleic acids. Proc Natl Acad Sci U S A.

[137] Wang G, Jing K, Balczon R, Xu X (2001). Defining the peptide nucleic acids (PNA) length requirement for PNA binding-induced transcription and gene expression. J Mol Biol.

[138] Zaghloul EM, Gissberg O, Moreno PMD, Siggens L, Hällbrink M, Jørgensen AS (2017). CTG repeat-targeting oligonucleotides for down-regulating Huntingtin expression. Nucleic Acids Res.

[139] Andrieu-Soler C, Casas M, Faussat A-M, Gandolphe C, Doat M, Tempé D (2005). Stable transmission of targeted gene modification using single-stranded oligonucleotides with flanking LNAs. Nucleic Acids Res.

[140] Knauert MP, Kalish JM, Hegan DC, Glazer PM (2006). Triplex-stimulated intermolecular recombination at a single-copy genomic target. Mol Ther.

[141] Chin JY, Kuan JY, Lonkar PS, Krause DS, Seidman MM, Peterson KR (2008). Correction of a splice-site mutation in the beta-globin gene stimulated by triplex-forming peptide nucleic acids. Proc Natl Acad Sci U S A.

[142] Chin JY, Reza F, Glazer PM (2013). Triplex-forming peptide nucleic acids induce heritable elevations in gamma-globin expression in hematopoietic progenitor cells. Mol Ther.

[143] Cromwell CR, Sung K, Park J, Krysler AR, Jovel J, Kim SK (2018). Incorporation of bridged nucleic acids into CRISPR RNAs improves Cas9 endonuclease specificity. Nat Commun.

[144] Hanvey JC, Peffer NJ, Bisi JE, Thomson SA, Cadilla R, Josey JA (1992). Antisense and antigene properties of peptide nucleic acids. Science.

[145] Pooga M, Soomets U, Hällbrink M, Valkna A, Saar K, Rezaei K (1998). Cell penetrating PNA constructs regulate galanin receptor levels and modify pain transmission in vivo. Nat Biotechnol.

[146] Fabbri E, Manicardi A, Tedeschi T, Sforza S, Bianchi N, Brognara E (2011). Modulation of the biological activity of microRNA-210 with peptide nucleic acids (PNAs). ChemMedChem.

[147] Brognara E, Fabbri E, Aimi F, Manicardi A, Bianchi N, Finotti A (2012). Peptide nucleic acids targeting miR-221 modulate p27Kip1 expression in breast cancer MDA-MB-231 cells. Int J Oncol.

[148] Ghidini A, Bergquist H, Murtola M, Punga T, Zain R, Strömberg R (2016). Clamping of RNA with PNA enables targeting of microRNA. Org Biomol Chem.

[149] Gasparello J, Manicardi A, Casnati A, Corradini R, Gambari R, Finotti A (2019). Efficient cell penetration and delivery of peptide nucleic acids by an argininocalix[4]arene. Sci Rep.

[150] Jain HV, Beaucage SL (2016). An amphipathic trans-acting phosphorothioate DNA element delivers uncharged PNA and PMO nucleic acid sequences in mammalian cells. Curr Protoc Nucleic Acid Chem.

[151] Chinnery PF, Taylor RW, Diekert K, Lill R, Turnbull DM, Lightowlers RN. Peptide nucleic acid delivery to human mitochondria. Gene Ther. 1999;6:1919–28. 10.1038/sj.gt.3301061 10637443

[152] Cutrona G, Carpaneto EM, Ulivi M, Roncella S, Landt O, Ferrarini M (2000). Effects in live cells of a c-myc anti-gene PNA linked to a nuclear localization signal. Nat Biotechnol.

[153] Sforza S, Tedeschi T, Calabretta A, Corradini R, Camerin C, Tonelli R (2010). A peptide nucleic acid embedding a pseudopeptide nuclear localization sequence in the backbone behaves as a peptide mimic. Eur J Org Chem.

[154] Roberts TC, Langer R, Wood MJA (2020). Advances in oligonucleotide drug delivery. Nat Rev Drug Discov.

[155] Sasso JM, Ambrose BJB, Tenchov R, Datta RS, Basel MT, DeLong RK (2022). The progress and promise of RNA medicine─an arsenal of targeted treatments. J Med Chem.

[156] Demidov VV, Potaman VN, Frank-Kamenetskii MD, Egholm M, Buchard O, Sönnichsen SH (1994). Stability of peptide nucleic acids in human serum and cellular extracts. Biochem Pharmacol.

[157] McMahon BM, Mays D, Lipsky J, Stewart JA, Fauq A, Richelson E (2002). Pharmacokinetics and tissue distribution of a peptide nucleic acid after intravenous administration. Antisense Nucleic Acid Drug Dev.

[158] Fluiter K, Frieden M, Vreijling J, Rosenbohm C, De Wissel MB, Christensen SM (2005). On the in vitro and in vivo properties of four locked nucleic acid nucleotides incorporated into an anti-H-Ras antisense oligonucleotide. ChemBioChem.

[159] Volpi S, Cancelli U, Neri M, Corradini R (2020). Multifunctional delivery systems for peptide nucleic acids. Pharmaceuticals (Basel).

[160] Ma X, Devi G, Qu Q, Toh D-FK, Chen G, Zhao Y (2014). Intracellular delivery of antisense peptide nucleic acid by fluorescent mesoporous silica nanoparticles. Bioconjug Chem.

[161] Krieg AM, Yi AK, Matson S, Waldschmidt TJ, Bishop GA, Teasdale R (1995). CpG motifs in bacterial DNA trigger direct B-cell activation. Nature.

[162] Vollmer J, Jepsen JS, Uhlmann E, Schetter C, Jurk M, Wader T (2004). Modulation of CpG oligodeoxynucleotide-mediated immune stimulation by locked nucleic acid (LNA). Oligonucleotides.

[163] Lange MJ, Burke DH, Chaput JC (2019). Activation of innate immune responses by a CpG oligonucleotide sequence composed entirely of threose nucleic acid. Nucleic Acid Ther.

[164] Cogoi S, Zorzet S, Rapozzi V, Géci I, Pedersen EB, Xodo LE (2013). MAZ-binding G4-decoy with locked nucleic acid and twisted intercalating nucleic acid modifications suppresses KRAS in pancreatic cancer cells and delays tumor growth in mice. Nucleic Acids Res.

[165] Kanwar JR, Roy K, Kanwar RK (2011). Chimeric aptamers in cancer cell-targeted drug delivery. Crit Rev Biochem Mol Biol.

[166] Ishige T, Itoga S, Matsushita K (2018). Locked nucleic acid technology for highly sensitive detection of somatic mutations in cancer. Adv Clin Chem.

[167] Falanga AP, Cerullo V, Marzano M, Feola S, Oliviero G, Piccialli G (2019). Peptide nucleic acid-functionalized adenoviral vectors targeting G-quadruplexes in the P1 promoter of Bcl-2 proto-oncogene: a new tool for gene modulation in anticancer therapy. Bioconjug Chem.

[168] Chen S-S, Tu X-Y, Xie L-X, Xiong L-P, Song J, Ye X-Q (2018). Peptide nucleic acids targeting mitochondria enhances sensitivity of lung cancer cells to chemotherapy. Am J Transl Res.

[169] Manicardi A, Gyssels E, Corradini R, Madder A (2016). Furan-PNA: a mildly inducible irreversible interstrand crosslinking system targeting single and double stranded DNA. Chem Commun (Camb).

[170] Hu L, Takezawa Y, Shionoya M (2022). Metal-mediated DNA base pairing of easily prepared 2-oxo-imidazole-4-carboxylate nucleotides. Chem Sci.

[171] Woodcock CB, Ulashchik EA, Poopeiko NE, Shmanai VV, Reich NO, Shchepinov MS (2016). Rational manipulation of DNA methylation by using isotopically reinforced cytosine. ChemBioChem.

[172] Korshun VA, Pestov NB, Nozhevnikova EV, Prokhorenko IA, Gontarev SV, Berlin YA (1996). Reagents for multiple non-radioactive labelling of oligonucleotides. Synth Commun.

[173] Liu L, Han L, Wu Q, Sun Y, Li K, Liu Y (2021). Multifunctional DNA dendrimer nanostructures for biomedical applications. J Mater Chem B.

[174] Alenaizan A, Barnett JL, Hud NV, Sherrill CD, Petrov AS (2021). The proto-nucleic acid builder: a software tool for constructing nucleic acid analogs. Nucleic Acids Res.

[175] Dragomir IS, Asandei A, Schiopu I, Bucataru IC, Mereuta L, Luchian T (2021). The nanopore-tweezing-based, targeted detection of nucleobases on short functionalized peptide nucleic acid sequences. Polymers (Basel).

[176] Shen W, De Hoyos CL, Migawa MT, Vickers TA, Sun H, Low A (2019). Chemical modification of PS-ASO therapeutics reduces cellular protein-binding and improves the therapeutic index. Nat Biotechnol.

[177] Lesiński W, Mnich K, Golińska AK, Rudnicki WR (2021). Integration of human cell lines gene expression and chemical properties of drugs for Drug Induced Liver Injury prediction. Biol Direct.

[178] Aguirre-Plans J, Piñero J, Souza T, Callegaro G, Kunnen SJ, Sanz F (2021). An ensemble learning approach for modeling the systems biology of drug-induced injury. Biol Direct.

[179] Liu A, Walter M, Wright P, Bartosik A, Dolciami D, Elbasir A (2021). Prediction and mechanistic analysis of drug-induced liver injury (DILI) based on chemical structure. Biol Direct.

