# Supplementary material for: Artificial genetic polymers against human pathologies
Source: Biol Direct. 2022 Dec 6;17:39. doi: 10.1186/s13062-022-00353-7 (PMC9727881; doi:10.1186/s13062-022-00353-7)
Supplement: Supplementary file 1 — Additional file 1. Clinical trials of oligonucleotide based therapeutics. [file 13062_2022_353_MOESM1_ESM.docx]

| **NCT Number** | **Phase** | **Status** | **Condition** | **Drug/Trade name** | **Modification** | **n,**  **number of participants** | |
| --- | --- | --- | --- | --- | --- | --- | --- |
| **Antisense Oligonucleotides (ASO)** | | | | | | | |
| NCT03070782 | II | Completed | Elevated lipoprotein | ISIS 681257 (Pelacarsen) | PSO | [1] | 286 |
| NCT03360747 | II | Completed | Familial Chylomicronemia syndrome | Vupanorsen/AKCEA-ANGPTL3-L Rx | PSO, MOE |  |  |
| NCT02406833 | I | Completed | Primary Open Angle Glaucoma | ISTH0036 | LNA  (3+3 LNA modified gapmer) |  | 13 |
| NCT01703988 | I/II | Completed | Spinal Muscular Atrophy | Nusinersen | PSO, MOE | **.** | 34 |
| NCT04862767 | I | Recruiting | Solid tumor | TASO-001 (OT-101) | PSO |  | 9 |
| NCT01120288 | I | Completed | Neoplasms, Liver metastasis | EZN-2968 | PSO, LNA |  | 10 |
| NCT00159250 | I/II | Completed | Duchenne Muscular Dystrophy | AVI-4658 (PMO) | PMO | [2] | 7 |
| NCT05276297 | II | Recruiting | Chronic Hepatitis B | GSK3228836 | MOE |  | 184 |
| NCT01159028 | I | Completed | Acute Leukemia, Myelodysplastic syndrome | BP1001 | P-ethoxy, ASO, |  | 60 |
| NCT00100672 | I | Completed | Neoplasms | Liposome Entrapped c-raf Antisense Oligonucleotide – Easy to Use (LErafAON-ETU) | PSO, liposomes |  | 40 |
| NCT00466583 | I | Completed | Carcinoma, Lymphoma | EZN-2968 | LNA |  | 52 |
| NCT00048321 | II | Completed | Rheumatoid Arthritis | ISIS 104838 | PSO |  | 62 |
| NCT04442295 | I/II | Recruiting | Dravet Syndrome | STK-001 - |  |  | 78 |
| NCT01829113 | II | Completed | Non-Squamous Non-Small Cell Lung Cancer | Apatorsen (OGX-427) | MOE |  | 155 |
| NCT03780257 | I/II | Completed | Retinal Disease | QR-421a |  |  | 20 |
| 3NCT00959868 | I | NA | Bladder Cancer | OGX-427 | MOE |  | 36 |
| NCT01563302 | I/II | Completed | Lymphoma, Advanced cancers, DLBCL | IONIS-STAT3Rx | Constrained ethyl nucleosides at 5’ and 3’ ends with Phosphorothioate backbone |  | 64 |
| NCT02900027 | I | Completed | Elevated Triglycerides | APOC-III-L-Rx | Ligand conjugated antisense drug |  | 56 |
| NCT00021749 | I/II | Completed | Chronic Lymphocytic Leukemia, CLL | Oblimerson sodium, G3139 | PSO |  | 40 |
| NCT00059813 | II | Completed | Recurrent Renal Cell Cancer | Oblimerson sodium | PSO |  | 41 |
| NCT03263507 | I | Completed | Healthy Volunteers | Donidalorsen | Ligand conjugated antisense oligonucleotide |  | 32 |
| NCT00736450 | NA | Terminated | Adult Diffuse Large Cell Lymphoma | G3139  Oblimersen sodium | PSO | . | 160 |
| NCT00445913 | I | Completed | Type 1 Diabetes | Diabetes-suppressive dendritic cell vaccine | PSO | [3] | 10 |
| NCT01040832 | II | Completed | Squamous Cell Carcinoma of the Head and Neck Cancer | EMD 1201081 | PSO | [4] | 107 |
| NCT00729053 | II | Completed | Renal cell carcinoma | IMO-2055 | Unmethylated CpG dinucleotide motifs |  | 92 |
| NCT00636155 | II | Terminated | Lymphoma, Small lymphocytic leukemia, | EL625 (Cenersen) |  |  | 20 |
| **Morpholino (ASO)** | | | | | | | |
| NCT00159250 | I/II | Completed | Duchenne Muscular Dystrophy | AVI-4658 | PMO | [2] | 7 |
| NCT00381433 | I | Completed | Encephalitis | AVI-4065 | PMO |  | 12 |
| NCT01353027 | I | Completed | Ebola Hemorrhagic Fever | AVI-6002 |  |  | 30 |
| NCT01375985 | I | Terminated | Influenza | AVI-7100 |  |  | 8 |
| NCT01353040 | I | Completed | Marburg Hemorrhagic Fever | AVI-6003 | PMO  Positive charges on selected subunits. |  | 30 |
| NCT01566877 | I | Completed | Marburg Hemorrhagic Fever | AVI-7288 | PMO  Positive charges on selected subunits. |  | 40 |
| NCT02740972 | II | Completed | Duchenne Muscular Dystrophy | NS-065/NCNP-01 | PMO  Positive charges on selected subunits. | . | 16 |
| NCT04004065 | II | Completed | Duchenne Muscular Dystrophy | SRP-5051 | Peptide conjugated |  | 60 |
| NCT05524883 | I/II | Recruiting | Duchenne Muscular Dystrophy | DYNE-251 | Antibody conjugate  PMO |  | 46 |
| NCT00655499 | II | Completed | Colorectal cancer | irinotecan hydrochloride |  |  | 65 |
| **siRNA** | | | | | | | |
| NCT04270760 | II | Active, not recruiting | Cardiovascular disease | Olpasiran | 2’-fluoro, 2’-methoxy substitutions |  | 294 |
| NCT01591356 | I | Recruiting | Advanced malignant solid neoplasm | EphA2-targeting DOPC-encapsulated siRNA |  |  | 76 |
|  |  |  |  |  |  |  |  |
| NCT04995536 | I | Recruiting | Malignant Lymphoma | CpG-STAT3 siRNA CAS3/SS3 |  |  | 18 |
| NCT00716014 | I | Completed | Pachyonychia Congenita | TD101 |  |  | 1 |
| NCT00672542 | I | Completed | Metastatic Melanoma, absence of CNS metastasis | Proteasome siRNA and tumor antigen RNA-transfected dendritic cells |  |  | 12 |
| NCT00257647 | NA | Completed | Chronic Myeloid Leukemia | SV40 vectors carrying siRNA |  |  | 25 |
| NCT00363714 | I/II | Completed | Age-Related Macular Degeneration, Choroidal Neovascularization | AGN211745 | 2’-O-Methyl groups |  | 26 |
| NCT00938574 | I | Completed | Advanced Solid Tumors | Atu027 | Liposomal formulation |  | 34 |
| NCT00395057 | II | Terminated | Age-Related Macular Degeneration, Choroid Neovascularization | AGN 211745, Ranibizumab 500µg | 2’-O-Methyl groups |  | 138 |
| NCT05196373 | I/II | Not Yet Recruiting | Hypertrophic Scar | STP705 |  |  | 50 |
| aNCT03934307 | I | Active/Not recruiting | Hypertension | ALN-AGT01 (zilebesiran) | 3’-Adenosine modification |  | 124 |
| NCT03946449 | II | Active/Not recruiting | Alpha 1 – antitrypsin deficiency | ARO-AAT (Fazirsiran) | PSO, 2’-O-akyl modifications |  | 16 |
| NCT04536688 | I | Completed | Autosomal dominant polycystic kidney disease | RGLS4326 | PSO, (S)-constrained ethyl, 2’-O-methyl, 2’-deoxy-2’-fluoro |  |  |
| NCT02314052 | I/II | Terminated | Hepatocellular carcinoma | DCR-MYC | Liposomal formulation |  | 21 |
| NCT01437007 | I | Completed | Colorectal, pancreatic, gastric, breast and ovarian carcinoma with hepatic metastases | TKM-080301 | Lipid nanoparticle formulation |  | 1 |
| NCT02227459 | I | Completed | Moderate to extensive hepatic fibrosis | ND-L02-s0201 | Lipid nanoparticle formulation |  | 25 |
| NCT00306904 | II | Completed | Diabetic Macular Edema | Bevasiranib |  |  | 48 |
| NCT05184127 | II | Completed | COVID-19 | MIR 19 ® |  |  | 156 |
| NCT00689065 | I | Terminated | Cancer | CALAA-01 | Nanoparticle formulation |  | 24 |
| NCT03108664 | III | Completed | Dry Eye Disease | SYL1001 |  |  | 330 |
| **Aptamers** | | | | | | | |
| NCT01505114 | II | Completed | HIV Infection | Tenofovir Disoproxil Fumarate | Multiple alkylation of purine nucleotides | [5] | 594 |
| NCT03362190 | II | Completed | Wet Age-Related Macular Degeneration | ARC1905 | 2’-F, PEG |  | 64 |
| NCT02397954 | II | Completed | Idiopathic Polypoidal Choroidal Vasculopathy | ARC1905 (Zimura) | 2’-F, PEG |  | 4 |
| NCT01089517 | II | Completed | Age-Related Macular Degeneration | E10030 | Fluorination and O-methylation of the 20^th^ nucleotide | [6] | 449 |
| NCT01547897 | II | Completed | Type 2 diabetes mellitus, Albuminuria | NOX-36 | PEG |  | 76 |
| NCT04901741 | II | Not yet recruiting | Metastatic Pancreatic cancer | NOX-A12 | PEG |  | 68 |
| NCT04121455 | I/II | Recruiting | Glioblastoma | NOX-A12 (Olaptesed pegol) | PEG |  | 27 |
| NCT02387957 | II | Completed | Age-Related Macular Degeneration | Fovista | PEG |  | 63 |
| NCT00113997 | I | Completed | Healthy | REG1 (anivamersen, pegnivacogin) | Guanosine 5’ modification  PEG |  | 106 |
| NCT02686658 | II/III | Completed | Geographic Atrophy, Macular Degeneration | Zimura | PEG |  | 286 |
| NCT00056199 | I | Completed | Hippel-Lindau Disease | EYE001(Pegaptanib) | PEG |  | 5 |
| NCT00215670 | II/III | Completed | Age-related Macular Degeneration | pegaptanib sodium (Macugen) | PEG | . | 125 |
| NCT00312351 | IV | Terminated | Macular Degeneration | Pegaptanib sodium | PEG |  | 262 |
| NCT00694785 | II | Withdrawn | Von Willebrand Disease | ARC1779 | PEG |  | 2 |
| NCT00632242 | II | Completed | Purpura, Thrombotic Thrombocytopenic Von Willebrand Disease Type-2b | ARC1779 | PEG |  | 28 |
| NCT04742062 | I | Completed | Stroke | ApTOLL |  |  | 46 |
| NCT04677803 | II | Completed | Von Willebrand Disease, Hemophilia A | BT200 | PEG |  | 26 |
| NCT02079896 | I/II | Completed | Anemia, End stage renal disease | Lexaptepid pegol (NOX-H94) | PEG |  | 33 |
| NCT04606602 | I | Recruiting | Elevated Lysophosphatidic acid | SLN360 | N-acetyl galactosamine conjugated |  | 88 |

**SUPPLEMENTAL REFERENCES**

1. Stiekema LCA, Prange KHM, Hoogeveen RM, Verweij SL, Kroon J, Schnitzler JG, et al. Potent lipoprotein(a) lowering following apolipoprotein(a) antisense treatment reduces the pro-inflammatory activation of circulating monocytes in patients with elevated lipoprotein(a). Eur Heart J. 2020;41:2262–71.

2. Kinali M, Arechavala-Gomeza V, Feng L, Cirak S, Hunt D, Adkin C, et al. Local restoration of dystrophin expression with the morpholino oligomer AVI-4658 in Duchenne muscular dystrophy: a single-blind, placebo-controlled, dose-escalation, proof-of-concept study. Lancet Neurol. 2009;8:918–28.

3. Phillips BE, Garciafigueroa Y, Engman C, Trucco M, Giannoukakis N. Tolerogenic Dendritic Cells and T-Regulatory Cells at the Clinical Trials Crossroad for the Treatment of Autoimmune Disease; Emphasis on Type 1 Diabetes Therapy. Front Immunol. 2019;10:148.

4. Ruzsa A, Sen M, Evans M, Lee LW, Hideghety K, Rottey S, et al. Phase 2, open-label, 1:1 randomized controlled trial exploring the efficacy of EMD 1201081 in combination with cetuximab in second-line cetuximab-naïve patients with recurrent or metastatic squamous cell carcinoma of the head and neck (R/M SCCHN). Invest New Drugs. 2014;32:1278–84.

5. Gulick RM, Wilkin TJ, Chen YQ, Landovitz RJ, Amico KR, Young AM, et al. Safety and Tolerability of Maraviroc-Containing Regimens to Prevent HIV Infection in Women: A Phase 2 Randomized Trial. Ann Intern Med. 2017;167:384–93.

6. Park EJ, Choi J, Lee KC, Na DH. Emerging PEGylated non-biologic drugs. Expert Opin Emerg Drugs. 2019;24:107–19.
